# Local Insect Availability Partly Explains Geographical Differences in Floral Visitor Assemblages of *Arum maculatum* L. (Araceae)

**DOI:** 10.3389/fpls.2022.838391

**Published:** 2022-03-08

**Authors:** Danae Laina, Eva Gfrerer, Valerie Scheurecker, Roman Fuchs, Marielle Schleifer, Carina Zittra, Rüdiger Wagner, Marc Gibernau, Hans Peter Comes, Anja C. Hörger, Stefan Dötterl

**Affiliations:** ^1^Department of Environment and Biodiversity, Paris Lodron University of Salzburg, Salzburg, Austria; ^2^Department of Functional and Evolutionary Ecology, University of Vienna, Vienna, Austria; ^3^Department of Limnology, University of Kassel, Kassel, Germany; ^4^Laboratory of Sciences for the Environment, CNRS – University of Corsica, Ajaccio, France

**Keywords:** local pollinator availability, pollinator limitation, specialization, generalization, reproductive success, *Arum maculatum*, Psychodidae, Sphaeroceridae

## Abstract

Geographical variation in abundance and composition of pollinator assemblages may result in variable selection pressures among plant populations and drive plant diversification. However, there is limited knowledge on whether differences in local visitor and pollinator assemblages are the result of site-specific strategies of plants to interact with their pollinators and/or merely reflect the pollinator availability at a given locality. To address this question, we compared locally available insect communities obtained by light-trapping with assemblages of floral visitors in populations of *Arum maculatum* (Araceae) from north vs. south of the Alps. We further investigated whether and how the abundance of different visitors affects plants’ female reproductive success and examined the pollen loads of abundant visitors. Local insect availability explained inter-regional differences in total visitor abundance, but only partly the composition of visitor assemblages. Northern populations predominantly attracted females of *Psychoda phalaenoides* (Psychodidae, Diptera), reflecting the high availability of this moth fly in this region. More generalized visitor assemblages, including other psychodid and non-psychodid groups, were observed in the south, where the availability of *P. phalaenoides*/Psychodidae was limited. Fruit set was higher in the north than in the south but correlated positively in both regions with the abundance of total visitors and psychodids; in the north, however, this relationship disappeared when visitor abundances were too high. High pollen loads were recorded on both psychodids and other Diptera. We demonstrate for the first time that the quantitative assessment of floral visitor assemblages in relation to locally available insect communities is helpful to understand patterns of geographical variation in plant–pollinator interactions. This combined approach revealed that geographical differences in floral visitors of *A. maculatum* are only partly shaped by the local insect availability. Potential other factors that may contribute to the geographical pattern of visitor assemblages include the region-specific attractiveness of this plant species to flower visitors and the population-specific behavior of pollinators.

## Introduction

It is widely recognized that geographical variation in abundance and composition of pollinator assemblages can impose variable selective pressures on floral phenotypes within and among plant species (Waser et al., 1996; Dodd et al., 1999; Johnson, 2010; Van der Niet and Johnson, 2012; Hernández-Hernández and Wiens, 2020). Indeed, geographical differences in pollinator assemblages are often associated with variation in floral traits related to pollination (e.g., advertisement, rewards, and morphology; Cosacov et al., 2014; Newman et al., 2015; Parker et al., 2018; Ishii et al., 2019), frequently resulting in different degrees of pollinator specialization (Bustamante et al., 2010), pollinator effectiveness, pollination success, and plant fitness (Campbell, 1987; Johnson and Bond, 1992; Gómez et al., 2009a; Moeller et al., 2012).

Variation in pollinator assemblages among populations is generally thought to correspond to local pollinator availability (Grant and Grant, 1965; Phillips et al., 2020), but may also result from site-specific differences in pollinator attraction (Majetic et al., 2009; Peakall et al., 2010). However, there is limited knowledge about the relative importance of local pollinator availability vs. site-specific pollination strategies in shaping the local abundance and composition of pollinator assemblages. This is mainly because local pollinator availability is usually estimated from presence/absence data of particular pollinators (Diaz and Kite, 2002; Moeller, 2006; Johnson and Raguso, 2016 but see also Sayers et al., 2020) or by comparing pollinator visitation rates among populations, assuming that visitation rates positively correlate with local pollinator abundance (e.g., Gómez et al., 2009b, 2014; Rech et al., 2018).

However, without further quantification of local insect availability, a high visitation rate observed for a particular pollinator species could reflect either local predominance of that species, or its specific attraction from a diverse pool of potential pollinators (Primack and Inouye, 1993; Johnson, 2010). Similarly, a low visitation rate at a given locality could indicate that pollinators are indeed locally scarce (Herrera, 1989) and plants are pollen-limited; if so, such a plant population would be expected to evolve toward less reliance on pollinators (e.g., spontaneous selfing; Fausto et al., 2001; Kalisz et al., 2004; Knight et al., 2005; Moeller, 2006) or switch to novel pollinators (Pattemore and Wilcove, 2012). Low visitation rates could also indicate that pollinators (even if abundant in the habitat) are not effectively attracted by the observed plant individuals (Herrera, 1989), and evolution toward increased attraction of these particular pollinators might be expected (Ashman, 2000; Knight et al., 2005; Trunschke et al., 2017). In either instance, low visitation rates result in plant populations that are pollinator-limited, but the evolutionary implications would be different. Nevertheless, up to now, studies that quantify local insect availability in relation to visitor assemblages are scarce (Sayers et al., 2020).

Here, we use the brood-site deceptive *Arum maculatum* (Araceae) to investigate the role of local insect availability (in terms of composition and abundance) in shaping visitor assemblages and female reproductive success (fruit set and number of seeds per fruit) of individual plants. This widespread perennial herb is ideal for such a study as it is one of the few European plant species that temporarily traps floral visitors in a floral chamber (Lack and Diaz, 1991; Kite, 1995; Bröderbauer et al., 2012; Szenteczki et al., 2021), from which they can be easily collected. *Arum maculatum* is mainly pollinated by psychodid moth flies (*Psychoda* spp. *sensu lato*, Psychodidae, Diptera; Lack and Diaz, 1991; Diaz and Kite, 2002; Chartier et al., 2011, 2013; Espíndola et al., 2011), while pollinator visitation patterns vary across the species’ range, especially between populations north vs. south of the Alps (Espíndola and Alvarez, 2011; Espíndola et al., 2011). More specifically, Espíndola et al. (2011) reported high floral visitor abundances consisting predominantly of female *Psychoda phalaenoides* in North–Central Europe but low visitor abundances composed mainly of male and female *P. grisescens* in the Mediterranean region. Little is known about the pollinator effectiveness of other insect taxa found in the floral chambers of *A. maculatum*, especially in Mediterranean populations (e.g., Chironomidae; Chartier et al., 2011; Espíndola et al., 2011; Gibernau, 2016 but see Diaz and Kite, 2002). Regardless, there are two main hypotheses to explain geographical differences in pollinator assemblages among populations of *A. maculatum*. The first states that local insect availability affects the composition and abundance of visiting insects (Chartier et al., 2013; Szenteczki et al., 2021). The second hypothesis posits that the observed geographical pattern of floral visitors can be explained by differential attraction of the two psychodid species. Specifically, it is assumed that *P. phalaenoides* is more attracted by populations located in North–Central Europe, while *P. grisescens* is more attracted by populations in the Mediterranean region (Espíndola et al., 2011; Gibernau, 2016). However, it is still unclear how the abundance and composition of pollinators are related to local insect availability and/or the plant’s site-specific strategies to attract pollinators.

We determined patterns of visitor assemblages in 11 populations of *A. maculatum* (six from north and five from south of the Alps), recorded and quantified the insect communities at each site using light trap catches, and determined the effect of floral visitors on individual fruit set and on the number of seeds per fruit. In addition, we quantified and compared the pollen loads among the main visiting insects (e.g., Psychodidae and other dipteran families) to determine whether non-psychodids can also carry pollen and act as potential pollinators. Specifically, we asked: (1) How do floral visitor assemblages of *A. maculatum* differ in their abundance and composition between northern and southern populations? (2) Is there a difference in local insect availability between northern and southern populations, and if so, can this explain inter-regional differences in visitor assemblages? (3) Is there a positive correlation, in each region, between the reproductive success of *A. maculatum* and the abundance of all or certain visitors that are trapped by this plant species? And (4) do non-psychodids carry pollen, and if so, is their pollen load comparable to that of psychodids, indicating they are potentially as efficient pollinators as psychodids? Based on previous studies (Espíndola et al., 2011; see above), we hypothesize that visitor assemblages of northern *A. maculatum* populations are dominated by female *P. phalaenoides*, while those of the southern populations are more diverse but less numerous in individuals. Such regional differences in visitor assemblages could reflect respective differences in insect availability and/or plant attractiveness. Finally, we expect a positive correlation of fruit set and seed number per fruit with the number of trapped psychodids, but not of other visitors as their pollen loads are supposedly lower.

## Materials and Methods

### Study Plant Species

The distributional range of *A. maculatum* covers most of Europe, extending eastward to northern Turkey and the western Caucasus (Boyce, 2006; Govaerts et al., 2020). Its preferential habitats include deciduous woodlands, hedgerows and other shaded areas, on various soils (Sowter, 1949). The spike-like inflorescence consists of a central elongated structure (spadix), whose upper part is sterile (appendix), while its lower part bears the flowers (Gibernau et al., 2004). Fertile female flowers (14–36 per inflorescence; Albre et al., 2003; Chartier and Gibernau, 2009; number of ovules per flower: 1–7; M. Gibernau, unpubl. res.) are found at the base, followed by hair-like sterile female flowers, fertile male flowers (85–155 per inflorescence; Chartier and Gibernau, 2009), and another set of hair-like sterile male flowers (Lack and Diaz, 1991; Gibernau et al., 2004; Chouteau et al., 2008). The spadix is surrounded by a modified bract (spathe), which forms a floral chamber at the level of the flowers (Sowter, 1949; Lack and Diaz, 1991; Gibernau et al., 2004). During the first day of anthesis, the stigmas become receptive and the spathe unfolds revealing the appendix. Concomitantly, the appendix heats up and emits a dung-like scent, as typical for the oviposition sites of putative main pollinators (moth flies, Psychodidae, Diptera; Bermadinger-Stabentheiner and Stabentheiner, 1995; Kite, 1995; Kite et al., 1998; Chartier et al., 2013). Pollinators are thus lured by chemical deception and trapped in the floral chamber for at least one night. On the next day, pollen is released and shed onto the insects while the spathe and sterile flowers wither, allowing the insects to escape (Lack and Diaz, 1991; Gibernau et al., 2004). A few months after pollination, the red berry-like fruits are retained in an infructescence, with usually up to five seeds per fruit (Sowter, 1949; Lack and Diaz, 1991).

### Study Populations and Sampling of Floral Visitors

**Figure 1:**
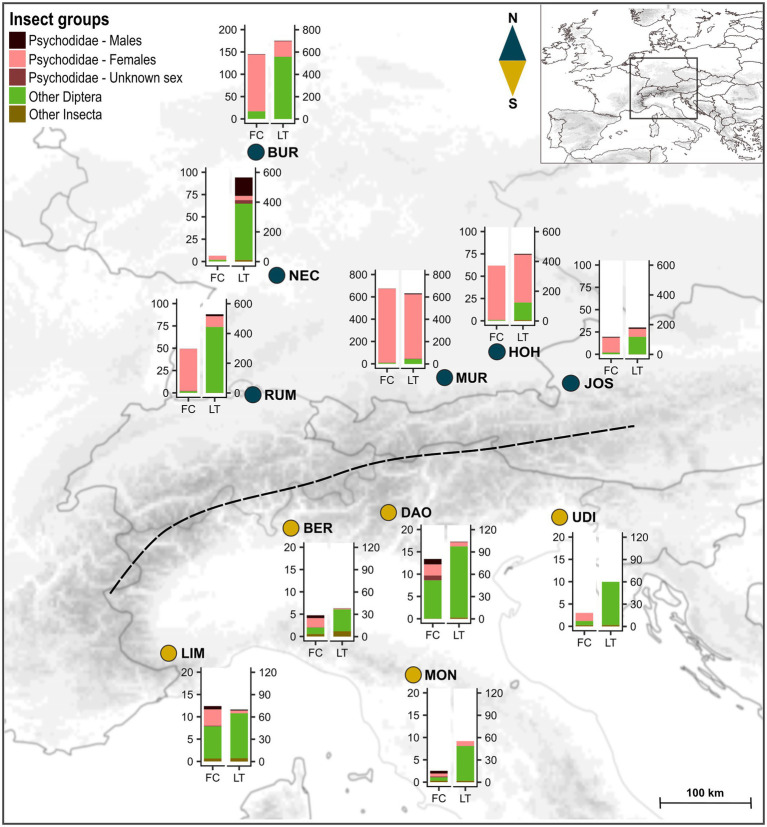
Localities of the sampled populations of *Arum maculatum* from north vs. south of the Alps (blue vs. dark yellow dots). The dashed line indicates the main chain of the Alps. Bar plots show the mean absolute abundances of insect groups recorded in the floral chambers (FC) and light trap catches per hour (LT) at each location. Note the differences in *y*-axis scales between the two regions and among northern populations. For sample sizes of plant individuals and light trap catches, see Supplementary Table S2. North: BUR, Burg Hohenstein; HOH, Hohendilching in Valley; JOS, Josefiau; MUR, Murnau am Staffelsee; NEC, Horb am Neckar; RUM, Rümikon; South: BER, Santa Maria Hoé; DAO, Daone; LIM, Limone Piemonte; MON, Montese; UDI, Udine. The map was prepared using the ETOPO1 Global Relief Model (Amante and Eakins, 2009) and ArcGIS ver. 10.4 (ESRI, Redland, CA, United States).

During three consecutive flowering seasons (April/May 2017–2019), we sampled floral visitors in 11 populations of *A. maculatum*, including six located north of the Alps (Austria, Germany, Switzerland: BUR, HOH, JOS, MUR, NEC, RUM) and five south of the Alps (North Italy: BER, DAO, LIM, MON, UDI; see Figure 1; Supplementary Table S1). These populations were located in shady habitats in the vicinity of (small) rivers and selected given their size and number of flowering individuals, with a minimum number of 30–40 inflorescences per population at the time of sampling (i.e., typically mid-flowering season). At most sites, we randomly selected 14–16 individuals (inflorescences), with a minimum distance of 1 m among each other to avoid sampling clones. Sample sizes were smaller in a northern population (HOH, *N* = 8) and higher in the largest northern (JOS, *N* = 71) and southern (DAO, *N* = 73) population (Figure 1; Supplementary Tables S1 and S2).

All insects trapped in the floral chambers were collected in the morning (08:00–10:00 h) of the second day of anthesis (i.e., before the release of the trapped insects), using insect-aspirators. After application of this non-invasive method, insects were placed in a preservative solution (96% ethanol, 4% glycerol) for later identification. We use the term “visitors” to describe all insects trapped in a floral chamber, regardless of their importance to pollination (see Discussion). Sampled plants were individually marked with metal tags, and infructescences were collected in summer, just before fruit ripening, to determine fruit set (see also Gfrerer et al., 2021) and the number of seeds per fruit.

### Assessment of Insect Availability at the Study Sites by Light Trap Catches

To determine insect abundance and community composition at each site, we carried out UV light-trapping (“Big Outback,” 12 V, Bioform, Nuremberg, Germany). The UV lamp was attached to a metal rod and surrounded by a net, from where attracted insects were collected using insect-aspirators. Light-trapping has been documented as an efficient method for the attraction of Psychodidae and other dipteran families (Withers, 1989; Borkent et al., 2018). The catches took place on the day(s) immediately after visitor sampling, between 20:00 and 23:00 h. This time period corresponds to the flowering time (female phase) of *A. maculatum* and the time of high activity of many (moth) fly species (Withers, 1989). At sites with a high insect availability (e.g., BUR, MUR, NEC, and RUM), *c*. 100 insects could be collected within 15–45 min. At sites with lower insect availability (e.g., MON and LIM), light trap catches were performed for up to 90 min in order to collect a similarly high number of insects. Light trap catches were carried out either once (most sites), twice (LIM) or three times (BER, DAO, JOS; see Results and Supplementary Tables S1 and S2). Since we were only interested in potential visitors of *A. maculatum*, we only caught light-attracted insects that were similar in size to those recorded in the floral chambers (*c.* 2–8 mm).

### Insect Identification

All insects sampled from the floral chambers and light traps were first sorted into Psychodidae vs. non-Psychodidae (Vaillant, 1971; Oosterbroek, 2006; Kvifte and Wagner, 2017), followed by sex determination of specimens belonging to Psychodidae. Whenever abdomens for sex determination were missing due to mechanical damage, specimens were categorized as “unknown sex (Psychodidae)”. Female psychodids were identified to species level according to morphological keys (Vaillant, 1971; Withers, 1989; Ježek, 1990; Svensson, 2009; Faucheux and Gibernau, 2011). In the present study, *Psychoda zetterstedti* refers to *P. albipennis* (its synonym; Ježek, 1983), which was previously recorded as a visitor in *A. maculatum* inflorescences (Espíndola et al., 2011). When species identification was not possible, individuals were categorized as “unidentified females (Psychodidae)”. All males of the same family were grouped under the category “males (Psychodidae)”, as further identification to species level was not possible. The remaining Diptera were identified to family level (Oosterbroek, 2006) and other insects to a higher taxonomic rank (Chinery, 1973). Unidentifiable specimens were grouped as “unidentified Diptera” or “unidentified Insecta”, respectively. In the following, insect categories (Supplementary Table S2) will be referred to as “insect groups”.

For our analyses of floral visitors and light trap catches, we only considered insect groups that occurred at least three times in floral chambers. Insects or other arthropods that occurred only once or twice (i.e., *Psychoda gemina*, *Philosepedon* spp., Phoridae, Hymenoptera, Lepidoptera, Arachnida, and Acarina), or were only recorded in light trap catches, were excluded from subsequent analyses. The same applied to rare visitors, which are very unlikely to pollinate *A. maculatum*, such as Collembola.

### Assessment of Pollen Loads

To determine the pollen loads of visiting insects, inflorescences of 45 individuals from JOS were enclosed in fine-mesh gauze bags early in the morning after the first day of anthesis. After escaping from the plants, all trapped insects were collected carefully from the bags, placed in glass vials and frozen. Pollen morphology (e.g., Weber et al., 1999) and the absence of other *Arum* species at this northern site allowed us to assign the pollen grains to *A. maculatum*. Pollen grain counting was carried out using a Leica DVM6 z-stacking digital microscope (Leica Microsystems, Wetzlar, Germany).

### Statistical Analyses

#### Insect Composition and Abundance in Floral Chambers and Light Trap Catches

Differences in the overall abundance of insect groups between northern and southern populations were determined separately for floral chambers (FC) and light trap catches (LT), using asymptotic Wilcoxon–Mann–Whitney (WMW) tests. For the JOS population, no LT data were available in 2018. To allow for comparison, the respective FC data were excluded from subsequent visitation analyses (Supplementary Table S1). Differences in insect composition between the two regions were again separately assessed for FC and LT, using permutational analysis of variance (PERMANOVA; 9,999 permutations) in PRIMER ver. 6.0 (Clarke and Gorley, 2005). For this analysis, pairwise Bray–Curtis similarities were calculated on the relative abundance of the insect groups (i.e., absolute abundance of each insect group divided by the total number of insects in a floral chamber or light trap). The term *region* was used as fixed factor, and we additionally included *population* nested in *region* as a random factor for stratification by population. As most of the southern populations were sampled in 2017 and most of the northern ones in 2018 (Supplementary Table S1), the term *year of sampling* was not included as a random factor due to the unbalanced sample sizes among years. Unidentified insect groups [i.e., “unidentified females (Psychodidae)”, “unknown sex (Psychodidae)”, “unidentified Diptera”, and “unidentified Insecta”; see Supplementary Table S2] were not considered for PERMANOVA.

**Figure 2:**
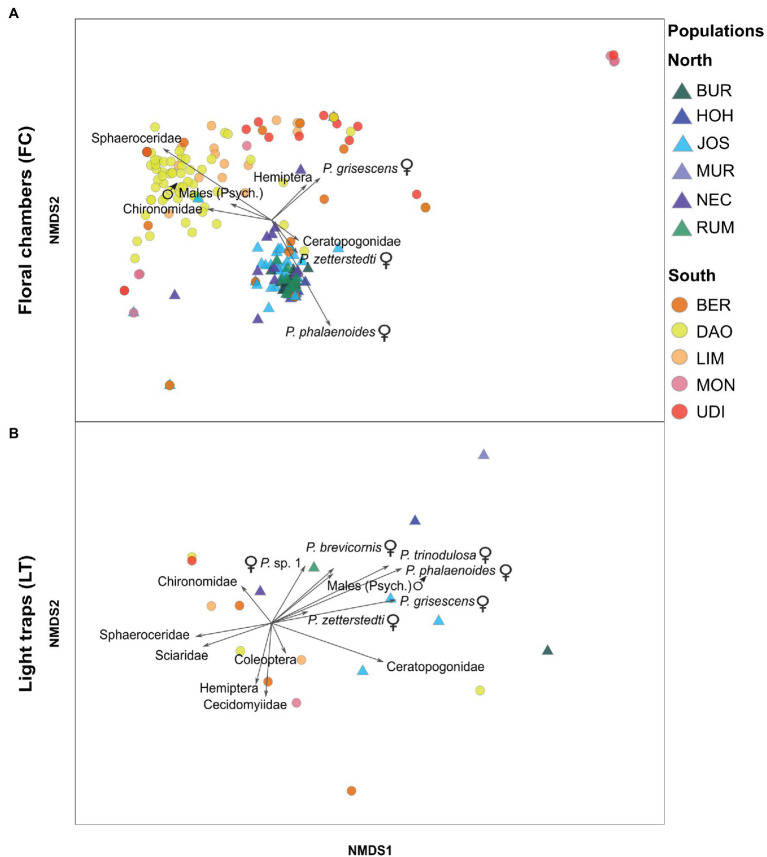
Non-metric multidimensional scaling (NMDS) based on Bray–Curtis similarities of relative abundances of insect groups in **(A)** floral chambers (FC) of *A. maculatum* (stress = .11) and **(B)** light trap (LT) catches (stress = 0.11), as recorded in populations from north (triangles) vs. south (circles) of the Alps (see Figure 1; Supplementary Table S1 for population code identification). Each point (circle or triangle) within the ordination space represents either a floral chamber **(A)** or a light trap **(B)**. Psych.: Psychodidae, *P*.: *Psychoda*.

The Bray–Curtis similarities were also used to visualize similarities and dissimilarities in the relative abundances of the insect groups among plant samples and among light trap catches, by employing non-metric multidimensional scaling (NMDS) in PRIMER. Vectors, representing Spearman’s rank correlations of individual insect groups with the ordination axes, were calculated. Only vectors with a correlation coefficient of at least 0.2 are reported. Insect groups that were strongly correlated with the ordination axes in the FC plot, and explained most of the variation observed between northern and southern FC-samples (see Results, Figure 2A), were retained for further analyses. Specifically, we tested whether the total abundance of each of these specific insect groups differed between the regions for FC and LT separately, using asymptotic WMW tests in the R package COIN (Hothorn et al., 2008; R Core Team, 2020).

**Figure 3:**
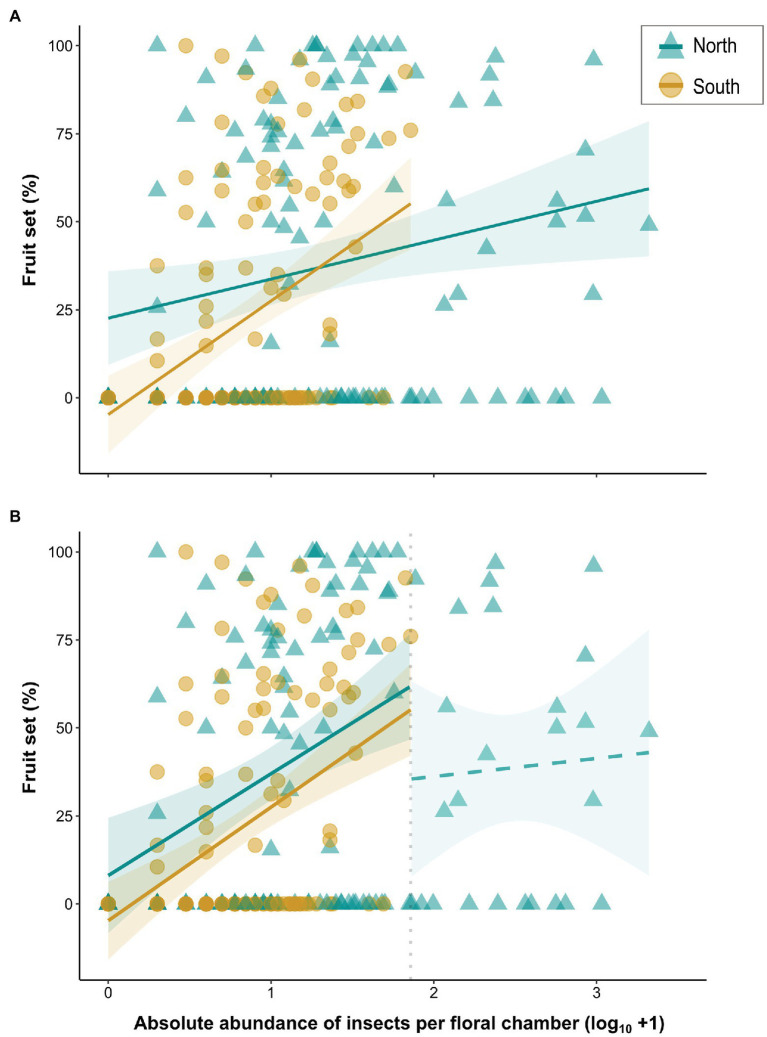
Absolute abundances (log_10_ + 1) of different insect groups most responsible for similarities and dissimilarities in relative floral visitor assemblages (see Figure 2A) in the floral chambers of *A. maculatum* (FC) and light trap catches per hour (LT), as recorded in populations from **(A)** north and **(B)** south of the Alps. Different small or capital letters indicate a significant difference between insect groups within regions for FC or LT, respectively, according to exact Wilcoxon–Mann–Whitney (WMW) *post hoc* tests (*p* < .05). Asterisks (*) indicate a significant difference between regions for each insect group, based on asymptotic WMW tests (*p* < .05). The bold horizontal line within each box represents the median of the distribution, and the lower and upper limits represent the 1st quartile and 3rd quartile. The two whiskers indicate the range of most extreme values if these are no more than 1.5 times the interquartile distance from the median (Tukey, 1977). Outliers are depicted as grey circles. Psych.: Psychodidae, *P*.: *Psychoda*.

For each data set (FC, LT), we also tested for differences in abundance among the different insect groups within each region (see Results, Figure 3), first using Kruskal–Wallis tests, followed by exact WMW tests for *post hoc* analyses in COIN. For the FC tests, both analyses were stratified by population. For the LT data, such stratification was not meaningful as for most populations only a single light trap catch was performed (Supplementary Tables S1 and S2). The *α*-levels obtained from *post hoc* analyses were adjusted using the Benjamini–Hochberg correction in R ver. 3.6.3 (R Core Team, 2020).

For most analyses described above, LT data were standardized to the number of insects per hour of trapping (except for PERMANOVA and NMDS that were performed using relative insect data; see above). For those populations where repeated light trap catches were carried out (i.e., BER, DAO, JOS, and LIM), mean insect numbers (per hour of trapping) of the replicate samples were used for statistical analyses.

#### Relationships Between Visitor Abundances and Fruit Set

**Figure 4:**
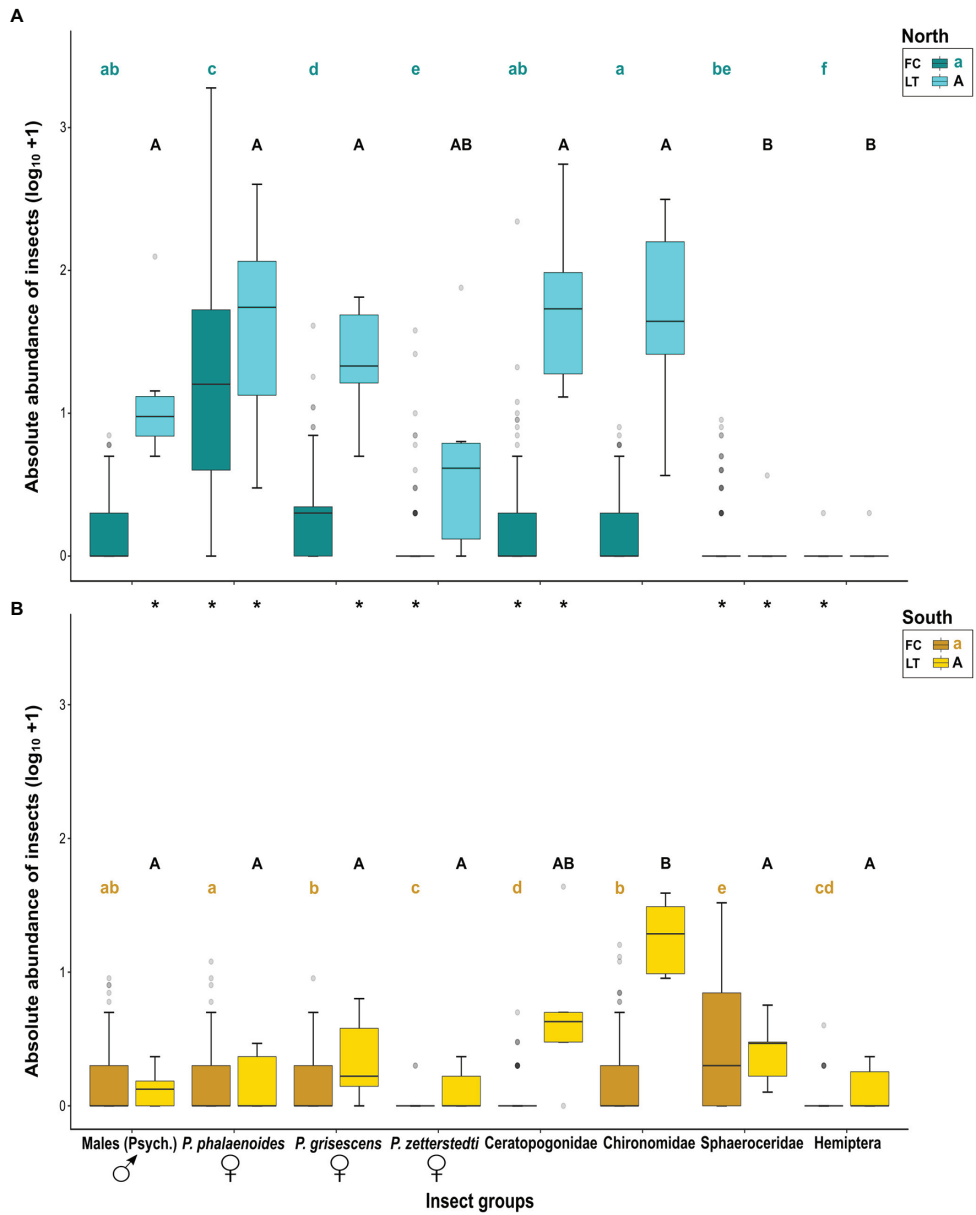
**(A)** Linear regressions between fruit set (%) of *A. maculatum* individuals and absolute abundance (log_10_ + 1) of visitors per floral chamber for populations from north (blue lines/triangles) vs. south (dark yellow lines/circles) of the Alps. **(B)** Linear regressions based on floral chambers with a maximum of 71 visitors, i.e., the highest number observed in the south (vertical dotted line; see text for details). The regression line beyond this threshold was calculated separately for the northern chambers that trapped more than 71 flies. Solid and dashed lines indicate significant and non-significant model fitting, respectively. Shaded areas indicate 95% confidence intervals (CIs) around regression lines.

Fruit set data were available for 260 out of the 273 plant individuals examined. First, we performed logistic regressions for the two regions to test whether the presence of an infructescence is dependent on the *total number of all floral visitors* (*sum*), using generalized linear models (GLMs) in R. Fruit set was then quantified as the percentage of fruits with seeds divided by the total number of female flowers. If no infructescence was available (*N* = 144 out of 260 individuals), fruit set was coded as zero. To assess whether there is a relationship between fruit set and the abundance of floral visitors north and south of the Alps, we constructed linear models (LMs) in R, including *fruit set* as response variable. In the full (across-region) model, the *total number of all floral visitors* (*sum*) (Supplementary Table S2) and *region* were incorporated as explanatory variables, testing also for their interaction. In addition, LMs were constructed separately for each region. In all three models, *population* was not included as random factor. This is because a significant random effect was only detected in the linear mixed effect model for the north after taking observations on visitor abundance above a defined threshold (“breakpoint”) into account (see below; R package LME4, Bates et al., 2015; R package RLRsim, Scheipl et al., 2008; Supplementary Table S4). Hence, the simpler model (i.e., without random effect) was preferred. The term *year of sampling* was not included as random factor due to the unbalanced sample sizes among years (see also above; Supplementary Table S1). For all models presented above (GLMs, LMs), insect counts were *log*-transformed (log_10_ + 1) to reduce the skewness in the data set. As various northern plants had higher visitor abundances than any from south of the Alps (see Results, Figure 4), we also examined the relationship between visitor abundance and fruit set by focusing only on those observations where the visitor-abundance values for the two regions overlapped. Therefore, we set a breakpoint at the maximum value of abundance observed in the south (i.e., maximum of 71 insects recorded in a floral chamber) and the analyses described above were repeated. As the interaction of the two variables (*sum* and *region*) proved non-significant (data not shown), no such term was employed in the models. For the north, observations with more than 71 insects were analyzed in a separate linear model.

To test for a relationship between the abundance of individual insect groups and fruit set, we constructed LMs for each region separately, including only the data up to the previously defined breakpoint. To assess whether there are differences in fruit set between the regions, we further constructed LMs for each insect group incorporating the term *region*. The analyses were performed for each major insect group (pooled psychodids, pooled non-psychodids; Supplementary Table S2) and other groups that contributed most to the floral visitation (see Results, Figure 2A). Insect species and groups with less than five observations per region (i.e., *Psychoda zetterstedti* and Hemiptera) were excluded.

#### Relationship Between Visitor Abundances and Number of Seeds Per Fruit

The number of seeds produced per fruit was calculated for each plant by dividing the total number of seeds by the number of fruits with seeds. Therefore, only individuals exhibiting an infructescence in summer (*N* = 116 out of 260 individuals) were included in this analysis. To test for a relationship between the number of seeds produced per fruit and the abundance of floral visitors, LMs were constructed as for the fruit set analyses in R, i.e., including a full (across region) model and one for each region separately. For the northern region, two separate models were additionally constructed with respect to the maximum visitor abundance observed in the south (breakpoint at 71 insects; see above). The number of seeds per fruit was used as response variable and the overall visitor abundance (*sum*) or the abundances of each individual insect group separately (see also above) as explanatory variables. The individual insect groups tested for the number of seeds per fruit were the same as in the fruit set analyses (listed in Supplementary Table S3).

#### Analysis of Pollen Loads

A Kruskal–Wallis test (R package COIN; Hothorn et al., 2008) was employed to test for differences in the number of pollen grains among insect groups, considering only those groups with more than five individuals.

## Results

### Total Absolute Abundances of Insects in Floral Chambers and Light Trap Catches North and South of the Alps

Across the 11 populations of *A. maculatum*, a total of 19,306 insects were collected, of which 16,296 were found in the floral chambers (*N* = 273) and 3,010 derived from light trap catches (*N* = 18). The abundance of floral visitors was quite variable in both regions, but significantly higher in northern plants (median = 16, range = 0–2,097) than southern ones (median = 6, range = 0–71; asymptotic WMW, *Z* = 5.41, *p* < .001, *N*_North_ = 139, *N*_South_ = 134; Figure 1; Supplementary Table S2; Supplementary Figure S1). Similarly, the abundance of insects trapped by light per hour was significantly higher in the northern region (median = 490, range = 55–701) compared to the south (median = 54, range = 6–158; asymptotic WMW, *Z* = 2.73, *p* = .006, *N*_North_ = 6, *N*_South_ = 5; Figure 1; Supplementary Table S2; Supplementary Figure S1).

### Relative Composition of Floral Visitors and Locally Available Insect Communities North and South of the Alps

#### Floral Chambers (FC)

In the northern populations, insect visitors collected from the floral chambers were mainly Psychodidae, while besides those, other dipteran families (e.g., Sphaeroceridae and Chironomidae) were the predominant visitors in the south (see below; Supplementary Table S2, Supplementary Figure S1). Within Psychodidae, females were the strongly dominating sex in the northern floral chambers (Figure 1; Supplementary Table S2). In the southern floral chambers, male psychodids were relatively more abundant compared to the north (Figure 1; Supplementary Table S2).

PERMANOVA revealed significant within-region (*pseudo-F*_9, 226_ = 6.26, *p* < .001) and among-region (*pseudo-F*_1, 226_ = 21.36, *p* = .002) differences in the relative abundances of the different insect groups found in the floral chambers. The latter difference was mainly due to the higher relative abundances of (1) female *Psychoda phalaenoides*, followed by *P. zetterstedti* and Ceratopogonidae in the north and (2) Sphaeroceridae, followed by various other groups (i.e., female *Psychoda grisescens*, Chironomidae, Hemiptera, male psychodids) in the south (see length and direction of vectors in Figure 2A).

#### Light Trap Catches (LT)

Light trap catches north of the Alps consisted mostly of Psychodidae and a few other dipteran families (e.g., Ceratopogonidae and Chironomidae), while the latter dominated the light trap catches in the south (Supplementary Table S2, Supplementary Figure S1). In one southern population (UDI), no psychodids were trapped. Within Psychodidae, females were dominant in most catches of both regions, with the exception of one population in the north (NEC, mostly males; Figure 1; Supplementary Table S2).

Similar to the floral chambers, there were significant among-region differences in the relative abundances of the different insect groups found in the light trap catches (PERMANOVA, *pseudo-F*_1, 7_ = 3.54, *p* = .017), whereas within-region differences were not detected (*pseudo-F*_9, 7_ = 1.21, *p* = .29). Northern light trap catches exhibited higher relative abundances of psychodid groups (e.g., females of *P. phalaenoides*, *P. grisescens*, and *P. trinodulosa*) compared to the southern catches (Figure 2B). In contrast, southern catches had higher relative abundances of non-psychodids, such as Sphaeroceridae, Sciaridae, Chironomidae, and Cecidomyiidae (Figure 2B).

### Absolute Abundances of Single Insect Groups in Floral Chambers and Light Trap Catches

#### North of the Alps

In the northern region, insect groups that were most responsible for similarities and dissimilarities in relative visitor assemblages among floral chambers (Figure 2A; see above) also showed significantly different absolute abundances across both floral chambers (Kruskal–Wallis, *χ*^2^ = 387.13, *df* = 7; *N* = 992, *p* < .001) and light traps (Kruskal–Wallis, *χ*^2^ = 30.19, *df* = 7, *N* = 48, *p* < .001). Floral chambers were dominated by female *P. phalaenoides* followed by female *P. grisescens* (Figure 3A; FC). The medians of absolute abundance of male psychodids, female *P. zetterstedti*, Ceratopogonidae, Chironomidae, Sphaeroceridae, and Hemiptera in the floral chambers were zero, while some representatives thereof (e.g., female *P. zetterstedti*) were found at high numbers in a few chambers (Figure 3A; FC). Male psychodids, female *P. phalaenoides*, female *P. grisescens*, Chironomidae, and Ceratopogonidae were attracted by the light traps in significantly higher numbers than Sphaeroceridae and Hemiptera, while the numbers of female *P. zetterstedti* were in between these two groups (Figure 3A; LT).

#### South of the Alps

In the southern region, the respective insect groups likewise differed from each other in their absolute abundances in both floral chambers (Kruskal–Wallis, *χ*^2^ = 222.46, *df* = 7, *N* = 1,072, *p* < .001) and light traps (Kruskal–Wallis, *χ*^2^ = 20.34, *df* = 7, *N* = 40, *p* = .005). In the southern floral chambers, however, Sphaeroceridae were the dominating insect group, followed by male psychodids, female *P. grisescens*, female *P. phalaenoides*, and Chironomidae (Figure 3B; FC). The remaining insect groups, i.e., female *P. zetterstedti*, Ceratopogonidae and Hemiptera, occurred in very few chambers (Figure 3B; FC). In contrast to the northern region, almost all insect groups found in the southern light traps exhibited similarly low abundances (Figure 3B; LT). As the only exception, Chironomidae were more abundant than all other insect groups, followed by Ceratopogonidae and the remaining insect groups (Figure 3B; LT).

#### Comparison Between Northern and Southern Regions

There was no significant difference between northern and southern floral chambers with regard to the total abundances of male psychodids and female *P. grisescens*, although higher numbers of these groups were attracted by the light at northern sites (see Figure 3). Female *P. phalaenoides* and Ceratopogonidae were significantly more abundant in the north than in the south, both in floral chambers and light traps; the opposite was true for Sphaeroceridae, which were significantly more abundant in the south compared to the north, both in floral chambers and light traps (Figure 3). Females of *P. zetterstedti* were more abundant in northern than southern chambers, but their numbers were similar in the light traps of each region (Figure 3). Although Hemiptera were generally low in abundance, they were more frequent in the southern chambers, but similarly abundant in the light traps of the two regions. Finally, Chironomidae exhibited similar numbers in the two regions, both in floral chambers and light traps with high abundances in the light traps and low abundances in floral chambers (Figure 3). Overall, these results indicate that differences in visitor assemblages within and between the two regions can only partly be explained by the light trap catches (see Discussion).

### Effects of Total Visitor Abundance on Fruit Set

Floral visitor abundance was a significant predictor of the presence of an infructescence in both regions, i.e., north (*p* = .004, *df* = 125; performance of the GLM, hereinafter referred to as GLM fit: *χ*^2^ = 9.26, *p* = .002) and south (*p* < .001, *df* = 131; GLM fit: *χ*^2^ = 19.35, *p* < .001) of the Alps (Supplementary Figure S2). Fruit set was highly variable (Figure 4) and the mean fruit set among the populations ranged between 1 and 86.1% in both of the regions (Supplementary Table S2; see also Gfrerer et al., 2021). When pooled across regions, the overall visitor abundance had a significant effect (slope = 11.06, *p* = .005) on fruit set (performance of the LM, hereinafter referred to as LM fit: adj. *R*^2^ = .13, *df* = 256, *p* < .001). Fruit set was significantly lower in the south than in the north [Δ(South–North) = −27.40, *p* = .002], whereby the slopes for the two regions also differed significantly, indicating a stronger effect of visitor abundance on fruit set in the south (Δslopes = 21.19, *p* = .009; Figure 4A). The LMs constructed separately for each region revealed a significant positive effect of the number of floral visitors on fruit set (higher insect numbers resulted in higher fruit set) in both the south (slope = 32.25, *p* < .001; LM fit: adj. *R*^2^ = .18, *df* = 131, *p* < .001; Figure 4A; Supplementary Table S3) and the north (slope = 11.06, *p* = .01; LM fit: adj. *R*^2^ = .04, *df* = 125, *p* = .01; Figure 4A).

When only considering floral chambers with a maximum of 71 insects (i.e., the highest number observed in the south; LM fit: adj. *R*^2^ = .18, *df* = 231, *p* < .001), fruit set was significantly lower, again, in the south [Δ(South–North) = −9.83, *p* = .03; Figure 4B], and the effect of visitor abundance (pooled across regions) on fruit set was also significant (slope = 30.51, *p* < .001). Interestingly, based on this analysis, although the total number of insects correlated positively with fruit set in the north (Figure 4B; Supplementary Table S3), no such correlation was detected in this region when only chambers with more than 71 insects were considered (LM fit: adj. *R*^2^ = −.04, *df* = 24, *p* = .78; Figure 4B).

### Effects of Individual Insect Groups on Fruit Set

**Figure 5:**
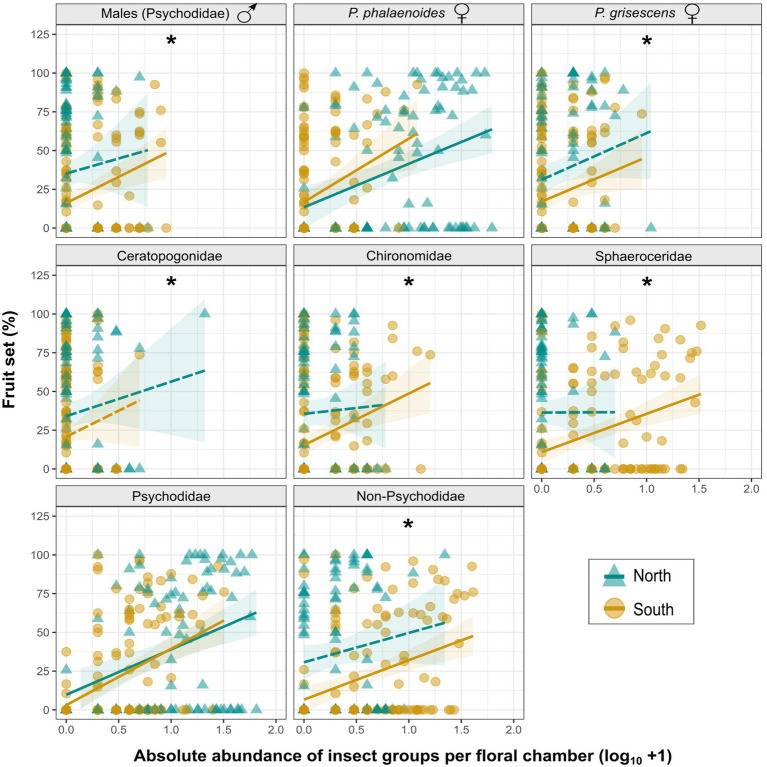
Linear regressions between fruit set (%) of *A. maculatum* individuals (see also Supplementary Table S3) and absolute abundance (log_10_ + 1) of different visitor groups (with a maximum of 71 visitors; see also Figure 4) per floral chamber for populations from north vs. south of the Alps (blue lines/triangles vs. dark yellow lines/circles). Psychodidae refer to the pooled psychodid groups and non-Psychodidae to the pooled non-psychodid groups. Solid and dashed lines indicate significant and non-significant model fitting, respectively. Shaded areas indicate 95% confidence intervals (CIs) around regression lines. Asterisks (*) indicate significant differences (*p* < .05) in fruit set between the two regions for each insect group.

When testing for relationships between the number of single insect groups and fruit set, significant effects (slopes) were detected in each of the two regions for (1) pooled Psychodidae (i.e., male psychodids, females of *Psychoda brevicornis*, *P. grisescens*, *P. phalaenoides*, *P.* sp. 1, *P. trinodulosa*, and *P. zetterstedti*, unidentified females, unknown sex; Supplementary Table S2); and (2) *P. phalaenoides* females (Figure 5; Supplementary Table S3). In the south, significant effects were additionally detected for (1) pooled non-psychodids (i.e., Cecidomyiidae, Ceratopogonidae, Chironomidae, Sciaridae, Sphaeroceridae, unidentified Diptera, Coleoptera, Hemiptera, and unidentified Insecta; Supplementary Table S2); (2) male psychodids; (3) *P. grisescens* females; (4) Chironomidae; and (5) Sphaeroceridae (Figure 5; Supplementary Table S3). Notably, pooled Psychodidae and female *P. phalaenoides* had the same effects on fruit set north and south of the Alps (no differences in the intercept), while for all other insect groups tested, the intercept of the model was higher in the north than in the south, that is, for a given number of individuals, the fruit set was higher in the north (Figure 5; Supplementary Table S3).

### Effects of the Abundance of All Visitors and of Individual Insect Groups on the Number of Seeds Per Fruit

The number of seeds per fruit varied in both regions (north: median = 1.12, range = 1–3.46; south: median = 1.18, range = 1–2.5), with no significant differences between the regions [Δ(South–North) = .03, *p* = .64; LM fit: adj. *R*^2^ = −.01, *df* = 113, *p* = .82]. Moreover, no correlation was detected between the abundance of floral visitors and the number of seeds per fruit both in the global LM (pooled across regions; slope = .03, *p* = .58) and in the LMs constructed separately for each region (north: slope = .08, *p* = .23; LM fit: *R*^2^ = .007, *df* = 63, *p* = .23; south: slope = −.16, *p* = .15; LM fit: *R*^2^ = .02, *df* = 49, *p* = .15; Supplementary Figure S3). Similar results were obtained when the same analysis was performed for the northern region, considering only chambers with either a maximum of 71 insects (slope = .18, *p* = .09; LM fit: *R*^2^ = .04, *df* = 47, *p* = .09) or more insects (slope = .21, *p* = .60; LM fit: *R*^2^ = −.05, *df* = 14, *p* = .60; Supplementary Figure S3). Furthermore, no correlation was detected between the abundance of individual insect groups and the number of seeds per fruit for both regions before the breakpoint (adj. *R*^2^ < .01, *p* > .05).

### Pollen Loads

**Figure 6:**
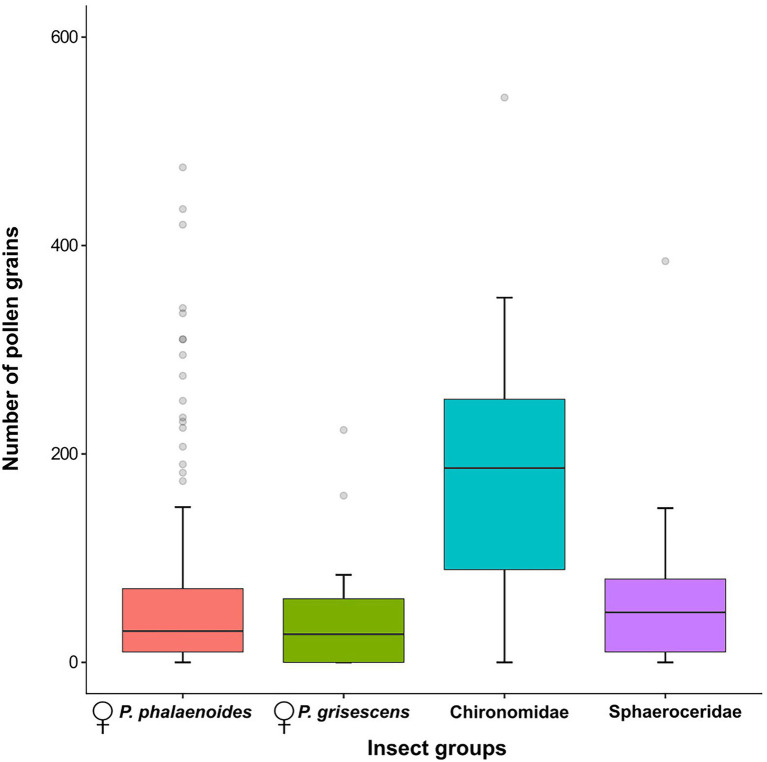
Number of pollen grains per individual for female *Psychoda phalaenoides* (*N* = 154), female *P. grisescens* (*N* = 17), Chironomidae (*N* = 8), and Sphaeroceridae (*N* = 9). The bold horizontal line within each box represents the median of the distribution, and the lower and upper limits represent the 1st quartile and 3rd quartile. The two whiskers indicate the range of most extreme values if these are no more than 1.5 times the interquartile distance from the median (Tukey, 1977). Outliers are shown as grey circles. Male psychodids, Ceratopogonidae, Sciaridae, and Coleoptera were not included in this figure because of their small sample sizes.

In total, 188 insect specimens were examined for pollen load (i.e., 154 females of *P. phalaenoides*, 17 females of *P. grisescens*, eight Chironomidae and nine Sphaeroceridae; Figure 6; Supplementary Figures S1, S4). Of those, 23 specimens carried no pollen grains (i.e., 15 females of *P. phalaenoides*, five females of *P. grisescens*, one Chironomidae and two Sphaeroceridae). Overall, the amount of pollen grains per individual was highly variable (range = 0–542). The median number of pollen grains was higher in Chironomidae (median = 187, range = 0–542) than in Sphaeroceridae (48, 0–385), *P. phalaenoides* (30, 0–475), and *P. grisescens* (27, 0–223); however, there were no significant differences in the number of pollen grains across these insect groups (Kruskal–Wallis, *χ*^2^ = 7.03, *df* = 3, *N* = 188, *p* = .07; Figure 6). Pollen grains were also detected on male psychodids (median = 18, range = 0–72, *N* = 4; Supplementary Figure S4), Ceratopogonidae (11, 0–47, *N* = 4), Sciaridae (8, 0–380, *N* = 3), and Coleoptera (10, 1–27, *N* = 3), but these groups were excluded from statistical analysis due to small sample sizes.

## Discussion

Based on our insect collections from floral chambers (Figure 1; Supplementary Figure S1), individuals of *A. maculatum* from north of the Alps attracted more visitors than southern ones. Moreover, the visitor assemblages of northern plants were strongly dominated by females of *Psychoda phalaenoides* (Figures 2A, 3A). By contrast, in the south, Sphaeroceridae were the most abundant insect visitors, followed by female *P. grisescens*, male psychodids, and Chironomidae (Figures 2A, 3B). When combined with our light trap results (Figures 2B, 3), these differences in visitor assemblages across the Alps could only be partially explained by the insects available in the different regions. Moreover, in each region, fruit set was quite variable, but significantly higher in the north than in the south and positively correlated with the total number of trapped insects as well as some, but not all specific visitor groups (Figures 4, 5; Supplementary Table S3). In the north, however, this positive relationship disappeared when numbers of trapped insects were too high (Figure 4B). The number of seeds per fruit did not differ between the regions and did not correlate with the overall abundance of floral visitors or with the abundance of individual insect groups (Supplementary Figure S3). Not only Psychodidae but also other fly taxa were found to carry high pollen loads, with amounts similar to those of psychodids (Figure 6).

### Does Local Insect Availability Shape the Visitor Assemblages of *Arum maculatum* Populations From North vs. South of the Alps?

The visitor assemblages we observed in *A. maculatum* populations from north vs. south of the Alps broadly concur with those reported by Espíndola et al. (2011), especially in terms of total and relative abundances of single psychodid species and the total abundance of other Diptera. For the southern region, however, we newly identified Sphaeroceridae as abundant visitors (Figures 2A, 3). This dipteran family has so far only been reported for a single northern (southwest German) population of *A. maculatum* (Roháček et al., 1990). That said, previous visitor collections from south of the Alps might well include specimens of Sphaeroceridae, yet classified as suborder Brachycera (Espíndola et al., 2011; Szenteczki et al., 2021). Regardless, the overall floral visitation patterns reported by Espíndola et al. (2011) are remarkably similar to those of the present study, suggesting spatial and temporal consistency in the regionally differing visitor assemblages across the Alps. It is noteworthy, however, that beyond our study region, Szenteczki et al. (2021) reported some temporal variation in floral visitors of *A. maculatum* populations in France, Croatia, and Serbia, after having re-surveyed those studied by Espíndola et al. (2011).

As evidenced by our light trap results, all psychodid groups, apart from female *P. zetterstedti*, exhibited similarly high and low abundances in the northern and southern regions, respectively (Figure 3; LT). Possible factors driving those inter-regional differences may include climatic conditions (Espíndola et al., 2011) and/or the availability of suitable habitats, which might be limited for these psychodid taxa in the south (R. Wagner, unpubl. res.). Further ecological investigations are needed to identify the underlying causes of the high and low abundances of Psychodidae north and south of the Alps, respectively.

Based on our comparative analyses of floral visitor and light trap catches (Figures 2, 3), local insect availability can partly explain the different visitor assemblages of *A. maculatum* from north vs. south of the Alps. Specifically, both the higher overall abundance of floral visitors and the higher absolute abundance of female *P. phalaenoides* north than south of the Alps can be explained by the locally available insects (Figures 2B, 3A). Similarly, the higher relative and absolute floral abundances of Sphaeroceridae in the south can be explained by their higher availability in this region (Figures 2B, 3B). These findings confirm a previous hypothesis by Chartier et al. (2013), proposing that local insect availability plays an important role in shaping the composition and abundance of floral visitors in *A. maculatum*.

However, the high relative abundances of female *P. phalaenoides* in the northern floral chambers (Figure 2A), and the similar absolute floral abundances of female *P. grisescens* and male psychodids in northern and southern populations (Figure 3), do not reflect the availability of these three insect groups in the respective regions. Instead, northern individuals of *A. maculatum* appear to be relatively more attractive to female *P. phalaenoides* than southern individuals, which preferentially trapped female *P. grisescens* and male psychodids (Figure 3). Our results therefore do not support the hypothesis that the relatively higher visitation of southern *A. maculatum* by female *P. grisescens* is due to the higher availability of this species (compared to *P. phalaenoides*) in the Mediterranean region (Espíndola et al., 2011). On the other hand, a putatively high attractiveness of northern *A. maculatum* to female *P. phalaenoides* inferred herein could explain why this specific interaction has been repeatedly observed in other northern locations of the plant’s distribution (Lack and Diaz, 1991; Diaz and Kite, 2002; Espíndola et al., 2011; Chartier et al., 2013; Gibernau, 2016). The recently reported differences in scent bouquets between *A. maculatum* populations from north vs. south of the Alps (Gfrerer et al., 2021) might play a key role in the differential attraction of the psychodid moth flies. To test this hypothesis, behavioral assays assessing whether these psychodid species respond differently to northern vs. southern scent bouquets are needed. Furthermore, it would be of interest to test whether the behavior of specific pollinator taxa differs among regions.

### Are Regional Differences in Plant Reproductive Success Caused by Variations in Pollinator Abundance, Effectiveness, and/or Resource Limitation?

The number of seeds per fruit was similar between the regions, but we observed lower levels of fruit set in southern than in northern populations of *A. maculatum* (Figure 4; Supplementary Table S2, Supplementary Figure S3). Taken together, these findings show that northern plants have a higher seed production than southern ones. The lower fruit set in the south is in agreement with the lower abundances of floral visitors in this region (Figures 1, 3, and 4; Supplementary Table S2). However, this inter-regional difference in fruit set is not due to differences in the numbers of insects trapped, as the same visitor abundance yielded higher fruit set in the north (Figure 4B). Possible explanations for this difference involve higher rates of fruit abortion in southern populations, which could be caused by stronger resource limitation (Uemura et al., 1993; Ollerton and Diaz, 1999; Suzuki, 2000; Albre et al., 2003) and/or bad weather conditions during flowering in this region (Kite, 1995; Wilcock and Neiland, 2002). Maternal effects might also differently affect the percentage of flowers setting fruit (Richardson and Stephenson, 1991) in the two regions. Furthermore, a relatively higher pollination effectiveness of northern, psychodid dominated, visitor assemblages could also explain this inter-regional difference in fruit set (Ollerton et al., 2007; Ne’eman et al., 2010; Willcox et al., 2017).

The latter hypothesis gains some support from the significant positive correlation observed between the abundance of pooled Psychodidae and fruit set in both regions (Figure 5; Supplementary Table S3), and the disappearance of significant inter-regional differences in fruit set when the numbers of trapped Psychodidae and female *P. phalaenoides* were similar between the regions (Figure 5; Supplementary Table S3). Both findings confirm the high pollination effectiveness of Psychodidae in *A. maculatum* (Lack and Diaz, 1991; Diaz and Kite, 2002); however, they also suggest that reduced fruit set in the south could be due to the lower floral abundance of psychodids there (Figure 5; Supplementary Table S3). Diaz and Kite (2002) reported that the high pollination effectiveness of *P. phalaenoides* could be related to the high amount of pollen carried, also by comparison with non-psychodid flies (e.g., Chironomidae: *Smittia pratorum*). Although we found no higher pollen loads on psychodids than non-psychodids (Figure 6), the former still might be the most efficient pollinators of *A. maculatum* due to certain behavioral characteristics (e.g., frequent movements around stigmas and less efficient cleaning behavior: Borkent and Schlinger, 2008; Ne’eman et al., 2010) or morphological traits (dense hairiness: Stavert et al., 2016).

Nonetheless, even if less effective, our results suggest that non-psychodids (e.g., Chironomidae and Sphaeroceridae) also contribute to the pollination of *A. maculatum* (Figure 5; Supplementary Table S3), as already demonstrated for Sphaeroceridae in Mediterranean *A. pictum* (Quilichini et al., 2010) and in an Australian aroid, *Typhonium eliosurum* (Sayers et al., 2020). This hypothesis is also supported by preliminary tests, where Sphaeroceridae were transferred from male to female stage inflorescences of *A. maculatum*, resulting in fruit set (M. Schleifer et al., unpubl. res.). Given the scarcity of Psychodidae in areas south of the Alps (Figures 1, 3; Supplementary Table S2), it is feasible that southern populations of *A. maculatum* evolved a more generalized pollination strategy by additionally attracting other, locally more abundant insect groups to counterbalance negative effects on fruit set (see also Bustamante et al., 2010; Hallett et al., 2017).

### Disappearance of the Positive Relationship Between Visitor Abundance and Fruit Set at Too High Visitor Numbers

The common assumption of a positive correlation between visitation rate and fruit set has recently been challenged (Sáez et al., 2014; Willcox et al., 2017). Sáez et al. (2014) found that overall fruit set in *Rubus idaeus* (Rosaceae) decreased with extremely high visitation rates of *Apis mellifera* and *Bombus terrestris* due to damage of the styles. In Araceae, we are aware of similar results only in *Dieffenbachia longispatha*, where fruit set peaked at intermediate abundances of pollinating beetles but decreased at both higher and lower abundances (Young, 1988). This was also explained by cumulative floral damage caused by the beetles’ activities (i.e., feeding and mating; Young, 1988). Although adult *Psychoda* flies lack feeding mouth parts (Oosterbroek, 2006), their activity inside the floral chamber of *A. maculatum* might also cause floral damage, for example, on the stigmatic papillae. Our finding (Figure 4B) might also be explained by an excess of pollen with a high number of simultaneously growing pollen tubes preventing each other from reaching the ovules (Ashman et al., 2004; Holland and Chamberlain, 2007).

## Conclusion

This study highlights how quantitative assessments of floral visitor assemblages in relation to locally available insect communities are helpful in understanding patterns of geographical variation in plant–pollinator interactions. To the best of our knowledge, this combined approach, which independently assesses floral visitation and local insect availability at each site, has never been applied before. We used light traps to quantify local insect availability, but other methods, such as Malaise or pitfall traps (Borkent et al., 2018; Sayers et al., 2020), need to be applied when working with strictly diurnal insects, or more generally, with pollinators that are not attracted by light sources. Consistent with previous studies, we found that psychodids are important pollinators of *A. maculatum*; however, our results also imply other insects, such as Sphaeroceridae and Chironomidae, as pollinators of this species. Future studies are needed to compare the pollination effectiveness of different visitors (psychodids and non-psychodids) in *A. maculatum*, and to better understand the species’ high variation in fruit set. Such studies would also reveal whether the lower fruit set observed in southern populations results from lower pollination effectiveness of southern visitor assemblages or other factors, such as resource limitation.

## Data Availability Statement

The raw data supporting the conclusions of this article will be made available by the authors upon request, without undue reservation.

## Author Contributions

SD, MG, ACH, and HPC designed the research. DL and EG conducted the fieldwork. DL and RW identified the psychodid specimens. VS and CZ identified the non-psychodid specimens. MS assessed the pollen loads. DL and RF performed the statistical analyses. DL wrote the first draft of the manuscript. All authors contributed to the article and approved the submitted version.

## Funding

This study was funded by a grant from FWF (Austrian Science Fund; P30175-B29) to ACH, HPC, and SD (PI). All sampling conducted comply with the current laws of the respective countries.

## Conflict of Interest

The authors declare that the research was conducted in the absence of any commercial or financial relationships that could be construed as a potential conflict of interest.

## Publisher’s Note

All claims expressed in this article are solely those of the authors and do not necessarily represent those of their affiliated organizations, or those of the publisher, the editors and the reviewers. Any product that may be evaluated in this article, or claim that may be made by its manufacturer, is not guaranteed or endorsed by the publisher.

## References

[ref2] Albre J. Quilichini A. Gibernau M. (2003). Pollination ecology of *Arum italicum* (Araceae). Bot. J. Linn. Soc. 141, 205–214. doi: 10.1046/j.1095-8339.2003.00139.x

[ref3] Amante C. Eakins B. W. (2009). ETOPO1 1 Arc-Minute Global Relief Model: Procedures, Data Sources and Analysis. NOAA Technical Memorandum NESDIS NGDC-24. Boulder: National Geophysical Data Center.

[ref5] Ashman T. L. (2000). Pollinator selectivity and its implications for the evolution of dioecy and sexual dimorphism. Ecology 81, 2577–2591. doi: 10.1890/0012-9658(2000)081[2577:PSAIIF]2.0.CO;2

[ref6] Ashman T. L. Knight T. M. Steets J. A. Amarasekare P. Burd M. Campbell D. R.. (2004). Pollen limitation of plant reproduction: ecological and evolutionary causes and consequences. Ecology 85, 2408–2421. doi: 10.1890/03-8024

[ref7] Bates D. Mächler M. Bolker B. M. Walker S. C. (2015). Fitting linear mixed-effects models using lme4. J. Stat. Softw. 67, 1–48. doi: 10.18637/jss.v067.i01

[ref8] Bermadinger-Stabentheiner E. Stabentheiner A. (1995). Dynamics of thermogenesis and structure of epidermal tissues in inflorescences of *Arum maculatum*. New Phytol. 131, 41–50. doi: 10.1111/j.1469-8137.1995.tb03053.x 33863164

[ref9] Borkent A. Brown B. V. Adler P. H. Amorim D. S. Barber K. Bickel D.. (2018). Remarkable fly (Diptera) diversity in a patch of costa Rican cloud forest: why inventory is a vital science. Zootaxa 4402, 53–90. doi: 10.11646/zootaxa.4402.1.3, PMID: 29690278

[ref10] Borkent C. J. Schlinger E. I. (2008). Pollen loads and pollen diversity on bodies of *Eulonchus tristis* (Diptera: Acroceridae): implications for pollination and flower visitation. Can. Entomol. 140, 257–264. doi: 10.4039/n07-061

[ref11] Boyce P. C. (2006). *Arum*: a decade of change. Aroideana 29, 132–137.

[ref12] Bröderbauer D. Diaz A. Weber A. (2012). Reconstructing the origin and elaboration of insect-trapping inflorescences in the Araceae. Am. J. Bot. 99, 1666–1679. doi: 10.3732/ajb.1200274, PMID: 22965851 PMC5608078

[ref13] Bustamante E. Casas A. Búrquez A. (2010). Geographic variation in reproductive success of *Stenocereus thurberi* (Cactaceae): effects of pollination timing and pollinator guild. Am. J. Bot. 97, 2020–2030. doi: 10.3732/ajb.1000071, PMID: 21616849

[ref14] Campbell D. R. (1987). Interpopulational variation in fruit production: the role of pollination-limitation in the Olympic Mountains. Am. J. Bot. 74, 269–273. doi: 10.1002/j.1537-2197.1987.tb08605.x

[ref15] Chartier M. Gibernau M. (2009). Size variations of flowering characters in *Arum maculatum* (Araceae). Aroideana 32, 153–158.

[ref16] Chartier M. Pélozuelo L. Buatois B. Bessière J. M. Gibernau M. (2013). Geographical variations of odour and pollinators, and test for local adaptation by reciprocal transplant of two European *Arum* species. Funct. Ecol. 27, 1367–1381. doi: 10.1111/1365-2435.12122

[ref17] Chartier M. Pélozuelo L. Gibernau M. (2011). Do floral odor profiles geographically vary with the degree of specificity for pollinators? Investigation in two sapromyophilous *Arum* species (Araceae). Ann. Soc. Entomol. Fr. 47, 71–77. doi: 10.1080/00379271.2011.10697698

[ref18] Chinery M. (1973). A Field Guide to the Insects of Britain and Northern Europe. 1st Edn. London: Collins Publishing.

[ref19] Chouteau M. Gibernau M. Barabé D. (2008). Relationships between floral characters, pollination mechanisms, life forms, and habitats in Araceae. Bot. J. Linn. Soc. 156, 29–42. doi: 10.1111/j.1095-8339.2007.00753.x

[ref20] Clarke K. R. Gorley R. (2005). Primer: Getting Started With v6. Plymouth: Primer-E Ltd.

[ref21] Cosacov A. Cocucci A. A. Sérsic A. N. (2014). Geographical differentiation in floral traits across the distribution range of the Patagonian oil-secreting *Calceolaria polyrhiza*: do pollinators matter? Ann. Bot. 113, 251–266. doi: 10.1093/aob/mct239, PMID: 24252281 PMC3890392

[ref22] Diaz A. Kite G. C. (2002). A comparison of the pollination ecology of *Arum maculatum* and *A. italicum* in England. Watsonia 24, 171–181.

[ref23] Dodd M. E. Silvertown J. Chase M. W. (1999). Phylogenetic analysis of trait evolution and species diversity variation among angiosperm families. Evolution 53, 732–744. doi: 10.1111/j.1558-5646.1999.tb05367.x, PMID: 28565649

[ref24] Espíndola A. Alvarez N. (2011). Comparative phylogeography in a specific and obligate pollination antagonism. PLoS One 6:e28662. doi: 10.1371/journal.pone.0028662, PMID: 22216104 PMC3246438

[ref25] Espíndola A. Pellissier L. Alvarez N. (2011). Variation in the proportion of flower visitors of *Arum maculatum* along its distributional range in relation with community-based climatic niche analyses. Oikos 120, 728–734. doi: 10.1111/j.1600-0706.2010.18937.x

[ref26] Faucheux M. J. Gibernau M. (2011). Antennal sensilla in five Psychodini mothflies (Diptera: Psychodidae: Psychodinae) pollinators of *Arum* spp. (Araceae). Ann. Soc. Entomol. Fr. 47, 89–100. doi: 10.1080/00379271.2011.10697700

[ref27] Fausto J. A. Eckhart V. M. Geber M. A. (2001). Reproductive assurance and the evolutionary ecology of self-pollination in *Clarkia xantiana* (Onagraceae). Am. J. Bot. 88, 1794–1800. doi: 10.2307/3558355, PMID: 21669612

[ref28] Gfrerer E. Laina D. Gibernau M. Fuchs R. Happ M. Tolasch T.. (2021). Floral scents of a deceptive plant are hyperdiverse and under population-specific phenotypic selection. Front. Plant Sci. 12:719092. doi: 10.3389/fpls.2021.719092, PMID: 34630465 PMC8500232

[ref29] Gibernau M. (2016). Pollinators and visitors of aroid inflorescences III: phylogenetic & chemical insights. Aroideana 39, 4–22.

[ref30] Gibernau M. Macquart D. Przetak G. (2004). Pollination in the genus *arum*: a review. Aroideana 27, 148–166.

[ref31] Gómez J. M. Abdelaziz M. Camacho J. P. M. Muñoz-Pajares A. J. Perfectti F. (2009a). Local adaptation and maladaptation to pollinators in a generalist geographic mosaic. Ecol. Lett. 12, 672–682. doi: 10.1111/j.1461-0248.2009.01324.x, PMID: 19453614

[ref32] Gómez J. M. Muñoz-Pajares A. J. Abdelaziz M. Lorite J. Perfectti F. (2014). Evolution of pollination niches and floral divergence in the generalist plant *Erysimum mediohispanicum*. Ann. Bot. 113, 237–249. doi: 10.1093/aob/mct186, PMID: 23965614 PMC3890381

[ref33] Gómez J. M. Perfectti F. Bosch J. Camacho J. P. M. (2009b). A geographic selection mosaic in a generalized plant-pollinator-herbivore system. Ecol. Monogr. 79, 245–263. doi: 10.1890/08-0511.1

[ref34] Govaerts R. Bogner J. Boos J. Boyce P. Cosgriff B. Croat T.. (2020). *World Checklist of Araceae (and Acoraceae)*. Kew: Royal Botanic Gardens. Available at: http://wcsp.science.kew.org/ (Accessed January 10, 2022).

[ref35] Grant V. Grant K. A. (1965). Flower Pollination in the Phlox Family. New York: Columbia University Press.

[ref36] Hallett A. C. Mitchell R. J. Chamberlain E. R. Karron J. D. (2017). Pollination success following loss of a frequent pollinator: the role of compensatory visitation by other effective pollinators. AoB Plants 9, 1–11. doi: 10.1093/aobpla/plx020 PMC5544916 28798863

[ref37] Hernández-Hernández T. Wiens J. J. (2020). Why are there so many flowering plants? A multi-scale analysis of plant diversification. Am. Nat. 195, 948–963. doi: 10.1086/708273, PMID: 32469653

[ref38] Herrera C. M. (1989). Pollinator abundance, morphology, and flower visitation rate: analysis of the “quantity” component in a plant-pollinator system. Oecologia 80, 241–248. doi: 10.1007/BF00380158, PMID: 28313114

[ref39] Holland J. N. Chamberlain S. A. (2007). Ecological and evolutionary mechanisms for low seed:ovule ratios: need for a pluralistic approach? Ecology 88, 706–715. doi: 10.1890/06-1283, PMID: 17503598

[ref40] Hothorn T. Hornik K. van de Wiel M. A. Zeileis A. (2008). Implementing a class of permutation tests: the coin package. J. Stat. Softw. 28, 1–23. doi: 10.18637/jss.v028.i0827774042

[ref41] Ishii H. S. Kubota M. X. Tsujimoto S. G. Kudo G. (2019). Association between community assemblage of flower colours and pollinator fauna: a comparison between Japanese and New Zealand alpine plant communities. Ann. Bot. 123, 533–541. doi: 10.1093/aob/mcy188, PMID: 30380008 PMC6377100

[ref42] Ježek J. (1983). Contribution to the taxonomy of the genus *Logima* Eat. (Diptera, Psychodidae). Acta Entomol. Musei Natl. Pragae 41, 213–234.

[ref43] Ježek J. (1990). Redescriptions of nine common palaearctic and holarctic species of Psychodini End. (Diptera: Psychodidae). Acta Entomol. Musei Natl. Pragae 43, 33–83.

[ref44] Johnson S. D. (2010). The pollination niche and its role in the diversification and maintenance of the southern African flora. Philos. Trans. R. Soc. B 365, 499–516. doi: 10.1098/rstb.2009.0243, PMID: 20047876 PMC2838267

[ref45] Johnson S. D. Bond W. J. (1992). Habitat dependent pollination success in a Cape orchid. Oecologia 91, 455–456. doi: 10.1007/BF00317637, PMID: 28313556

[ref46] Johnson S. D. Raguso R. A. (2016). The long-tongued hawkmoth pollinator niche for native and invasive plants in Africa. Ann. Bot. 117, 25–36. doi: 10.1093/aob/mcv137, PMID: 26346719 PMC4701141

[ref47] Kalisz S. Vogler D. W. Hanley K. M. (2004). Context-dependent autonomous self-fertilization yields reproductive assurance and mixed mating. Nature 430, 884–887. doi: 10.1038/nature02776, PMID: 15318220

[ref48] Kite G. C. (1995). The floral odour of *Arum maculatum*. Biochem. Syst. Ecol. 23, 343–354. doi: 10.1016/0305-1978(95)00026-Q

[ref49] Kite G. C. Hetterscheid W. L. A. Lewis J. M. Boyce P. C. Ollerton J. Cocklin E.. (1998). “Inflorescence odours and pollinators of *Arum* and *Amorphophallus* (Araceae),” in Reproductive Biology. eds. Owens S. J. Rudall P. J. (Royal Botanic Gardens: Kew), 295–315.

[ref50] Knight T. M. Steets J. A. Vamosi J. C. Mazer S. J. Burd M. Campbell D. R.. (2005). Pollen limitation of plant reproduction: pattern and process. Annu. Rev. Ecol. Evol. Syst. 36, 467–497. doi: 10.1146/annurev.ecolsys.36.102403.115320

[ref51] Kvifte G. M. Wagner R. (2017). “Chapter 24: Psychodidae (sand flies, moth flies or owl flies),” in Manual of Afrotropical Diptera. eds. Kirk-Spriggs A. H. Sinclair B. J. (Pretoria: South African National Biodiversity Institute), 607–632.

[ref52] Lack A. J. Diaz A. (1991). The pollination of *Arum maculatum* L. – a historical review and new observations. Watsonia 18, 333–342.

[ref53] Majetic C. J. Raguso R. A. Ashman T. L. (2009). The sweet smell of success: floral scent affects pollinator attraction and seed fitness in *Hesperis matronalis*. Funct. Ecol. 23, 480–487. doi: 10.1111/j.1365-2435.2008.01517.x

[ref54] Moeller D. A. (2006). Geographic structure of pollinator communities, reproductive assurance, and the evolution of self-pollination. Ecology 87, 1510–1522. doi: 10.1890/0012-9658(2006)87[1510:GSOPCR]2.0.CO;2, PMID: 16869427

[ref55] Moeller D. A. Geber M. A. Eckhart V. M. Tiffin P. (2012). Reduced pollinator service and elevated pollen limitation at the geographic range limit of an annual plant. Ecology 93, 1036–1048. doi: 10.1890/11-1462.1, PMID: 22764490

[ref56] Ne’eman G. Jürgens A. Newstrom-Lloyd L. Potts S. G. Dafni A. (2010). A framework for comparing pollinator performance: effectiveness and efficiency. Biol. Rev. 85, 435–451. doi: 10.1111/j.1469-185X.2009.00108.x 20015317

[ref57] Newman E. Manning J. Anderson B. (2015). Local adaptation: mechanical fit between floral ecotypes of *Nerine humilis* (Amaryllidaceae) and pollinator communities. Evolution 69, 2262–2275. doi: 10.1111/evo.12736, PMID: 26194119

[ref58] Ollerton J. Diaz A. (1999). Evidence for stabilising selection acting on flowering time in *Arum maculatum* (Araceae): the influence of phylogeny on adaptation. Oecologia 119, 340–348. doi: 10.1007/s004420050794, PMID: 28307756

[ref59] Ollerton J. Killick A. Lamborn E. Watts S. Whiston M. (2007). Multiple meanings and modes: on the many ways to be a generalist flower. Taxon 56, 717–728. doi: 10.2307/25065855

[ref60] Oosterbroek P. (2006). The European Families of the Diptera: Identification-Diagnosis-Biology. 2nd Edn. Utrecht: KNNV Publishing.

[ref61] Parker A. J. Williams N. M. Thomson J. D. (2018). Geographic patterns and pollination ecotypes in *Claytonia virginica*. Evolution 72, 202–210. doi: 10.1111/evo.13381, PMID: 29055134

[ref62] Pattemore D. E. Wilcove D. S. (2012). Invasive rats and recent colonist birds partially compensate for the loss of endemic New Zealand pollinators. Proc. R. Soc. B Biol. Sci. 279, 1597–1605. doi: 10.1098/rspb.2011.2036, PMID: 22090388 PMC3282350

[ref63] Peakall R. Ebert D. Poldy J. Barrow R. A. Francke W. Bower C. C.. (2010). Pollinator specificity, floral odour chemistry and the phylogeny of Australian sexually deceptive *Chiloglottis* orchids: implications for pollinator-driven speciation. New Phytol. 188, 437–450. doi: 10.1111/j.1469-8137.2010.03308.x, PMID: 20561345

[ref64] Phillips R. D. Peakall R. van der Niet T. Johnson S. D. (2020). Niche perspectives on plant–pollinator interactions. Trends Plant Sci. 25, 779–793. doi: 10.1016/j.tplants.2020.03.009, PMID: 32386827

[ref65] Primack R. B. Inouye D. W. (1993). Factors affecting pollinator visitation rates: a biogeographic comparison. Curr. Sci. 65, 257–262.

[ref66] Quilichini A. Macquart D. Barabé D. Albre J. Gibernau M. (2010). Reproduction of the West Mediterranean endemic *Arum pictum* (Araceae) on Corsica. Plant Syst. Evol. 287, 179–187. doi: 10.1007/s00606-010-0312-3

[ref67] R Core Team (2020). *R: A Language and Environment for Statistical Computing*. Vienna: R Foundation for Statistical Computing. Available at: https://www.R-project.org/ (Accessed May 17, 2021).

[ref68] Rech A. R. Jorge L. R. Ollerton J. Sazima M. (2018). Pollinator availability, mating system and variation in flower morphology in a tropical savanna tree. Acta Bot. Bras. 32, 462–472. doi: 10.1590/0102-33062018abb0220

[ref69] Richardson T. E. Stephenson A. G. (1991). Effects of parentage, prior fruit set and pollen load on fruit and seed production in *Campanula americana* L. Oecologia 87, 80–85. doi: 10.1007/BF00323783, PMID: 28313355

[ref70] Roháček J. Beck-Haug I. Dobat K. (1990). Sphaeroceridae associated with flowering *Arum maculatum* (Araceae) in the vicinity of Tübingen, SW-Germany. Senckenberg. Biol. 71, 259–268.

[ref71] Sáez A. Morales C. L. Ramos L. Y. Aizen M. A. (2014). Extremely frequent bee visits increase pollen deposition but reduce drupelet set in raspberry. J. Appl. Ecol. 51, 1603–1612. doi: 10.1111/1365-2664.12325

[ref72] Sayers T. D. J. Steinbauer M. J. Farnier K. Miller R. E. (2020). Dung mimicry in *Typhonium* (Araceae): explaining floral trait and pollinator divergence in a widespread species complex and a rare sister species. Bot. J. Linn. Soc. 193, 375–401. doi: 10.1093/botlinnean/boaa021

[ref73] Scheipl F. Greven S. Kuechenhoff H. (2008). Size and power of tests for a zero random effect variance or polynomial regression in additive and linear mixed models. Comput. Stat. Data Anal. 52, 3283–3299. doi: 10.1016/j.csda.2007.10.022

[ref74] Sowter F. (1949). *Arum maculatum* L. J. Ecol. 37, 207–219. doi: 10.2307/2256754

[ref75] Stavert J. R. Liñán-Cembrano G. Beggs J. R. Howlett B. G. Pattemore D. E. Bartomeus I. (2016). Hairiness: the missing link between pollinators and pollination. PeerJ 4:e2779. doi: 10.7717/peerj.2779, PMID: 28028464 PMC5180583

[ref76] Suzuki N. (2000). Pollinator limitation and resource limitation of seed production in the scotch broom, *Cytisus scoparius* (Leguminosae). Plant Species Biol. 15, 187–193. doi: 10.1046/j.1442-1984.2000.00038.x

[ref77] Svensson B. W. (2009). Fjärilsmyggfaunan i ett hagmarksområde och en ladugård i östra Blekinges skogsland. Med en översikt av familjen Psychodidae:s morfologi, systematik och utforskande, samt särskilt de svenska *Psychoda* s.l. -arternas biologi. Entomol. Tidskr. 130, 185–208.

[ref78] Szenteczki M. A. Godschalx A. L. Galmán A. Espíndola A. Gibernau M. Alvarez N.. (2021). Spatial and temporal heterogeneity in pollinator communities maintains within-species floral odour variation. Oikos 130, 1487–1499. doi: 10.1111/oik.08445

[ref79] Trunschke J. Sletvold N. Ågren J. (2017). Interaction intensity and pollinator-mediated selection. New Phytol. 214, 1381–1389. doi: 10.1111/nph.14479, PMID: 28240377

[ref80] Tukey J. W. (1977). Exploratory Data Analysis. 1st Edn. Reading: Addison-Wesley Publishing.

[ref81] Uemura S. Ohkawara K. Kudo G. Wada N. Higashi S. (1993). Heat-production and cross-pollination of the Asian skunk cabbage *Symplocarpus renifolius* (Araceae). Am. J. Bot. 80, 635–640. doi: 10.1002/j.1537-2197.1993.tb15233.x

[ref82] Vaillant F. (1971). “9d. Psychodidae – Psychodinae” in Die Fliegen der palaearktischen Region. ed. Lindner E. (Stuttgart: Schweizerbart’sche Verlagsbuchhandlung), 1–48.

[ref83] Van der Niet T. Johnson S. D. (2012). Phylogenetic evidence for pollinator-driven diversification of angiosperms. Trends Ecol. Evol. 27, 353–361. doi: 10.1016/j.tree.2012.02.002, PMID: 22445687

[ref84] Waser N. M. Chittka L. Price M. V. Williams N. M. Ollerton J. (1996). Generalization in pollination systems, and why it matters. Ecology 77, 1043–1060. doi: 10.2307/2265575

[ref85] Weber M. Halbritter H. Hesse M. (1999). The basic pollen wall types in Araceae. Int. J. Plant Sci. 160, 415–423. doi: 10.1086/314122

[ref86] Wilcock C. Neiland R. (2002). Pollination failure in plants: why it happens and when it matters. Trends Plant Sci. 7, 270–277. doi: 10.1016/S1360-1385(02)02258-6, PMID: 12049924

[ref87] Willcox B. K. Aizen M. A. Cunningham S. A. Mayfield M. M. Rader R. (2017). Deconstructing pollinator community effectiveness. Curr. Opin. Insect. Sci. 21, 98–104. doi: 10.1016/j.cois.2017.05.012, PMID: 28822496

[ref88] Withers P. (1989). Moth Flies. Diptera: Psychodidae. 1st Edn. Sheffield: Derek Whiteley.

[ref89] Young H. J. (1988). Differential importance of beetle species pollinating *Dieffenbachia longispatha* (Araceae). Ecology 69, 832–844. doi: 10.2307/1941033

