# Supplementary material for: Local Insect Availability Partly Explains Geographical Differences in Floral Visitor Assemblages of Arum maculatum L. (Araceae)
Source: Front Plant Sci. 2022 Mar 8;13:838391. doi: 10.3389/fpls.2022.838391 (PMC8957888; doi:10.3389/fpls.2022.838391)
Supplement: Supplementary file 1 [file Data_Sheet_1.doc]

Supplementary Material

# Supplementary Figures and Tables

## Supplementary Figures


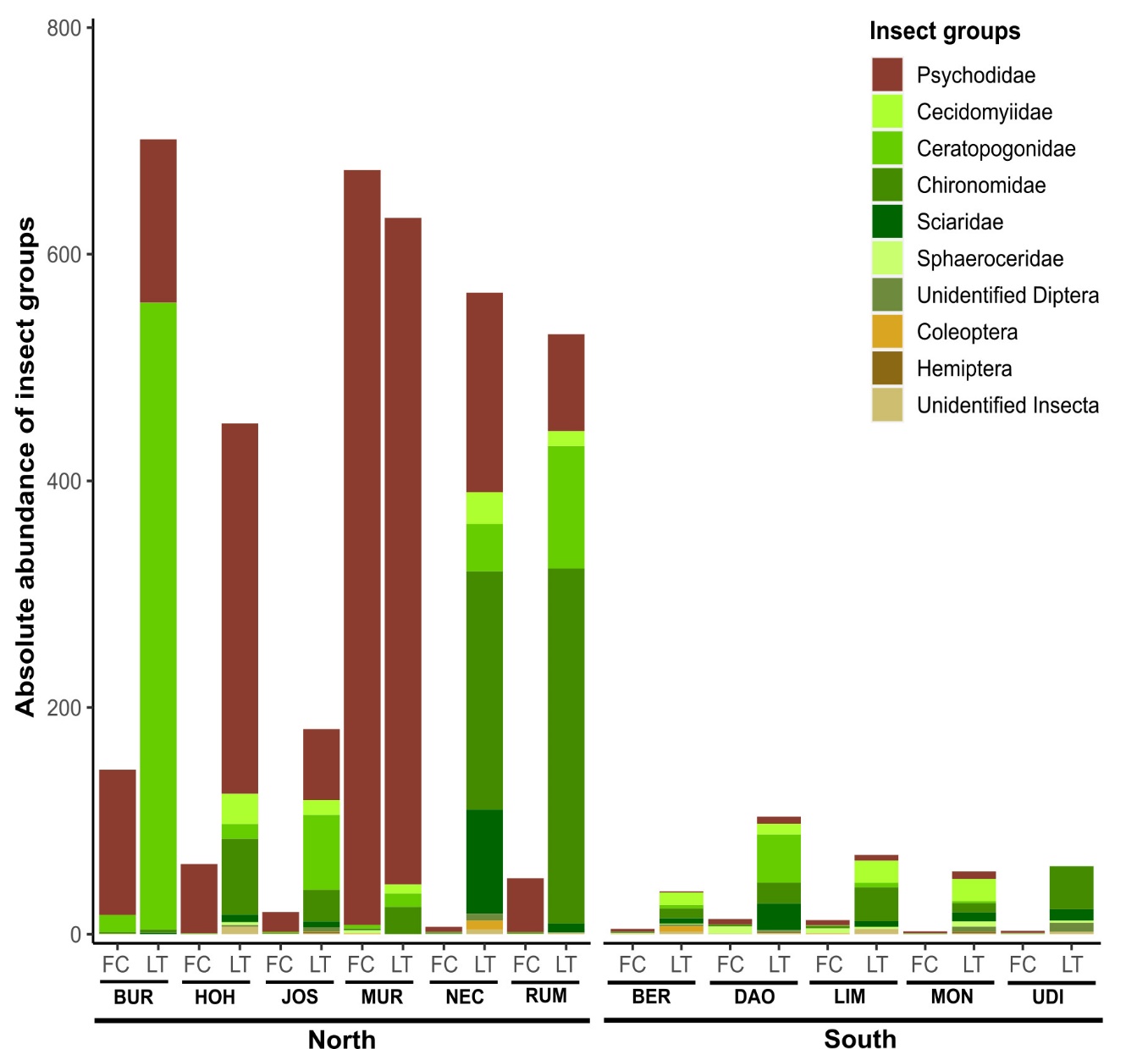


**Supplementary Figure 1.** Absolute abundances of dipteran families and other insect groups (see also **Supplementary Table** **S2**) recorded in floral chambers (FC) and light traps per hour (LT) for each population of *Arum maculatum* from north and south of the Alps (see **Supplementary Table S1** for population code identification).


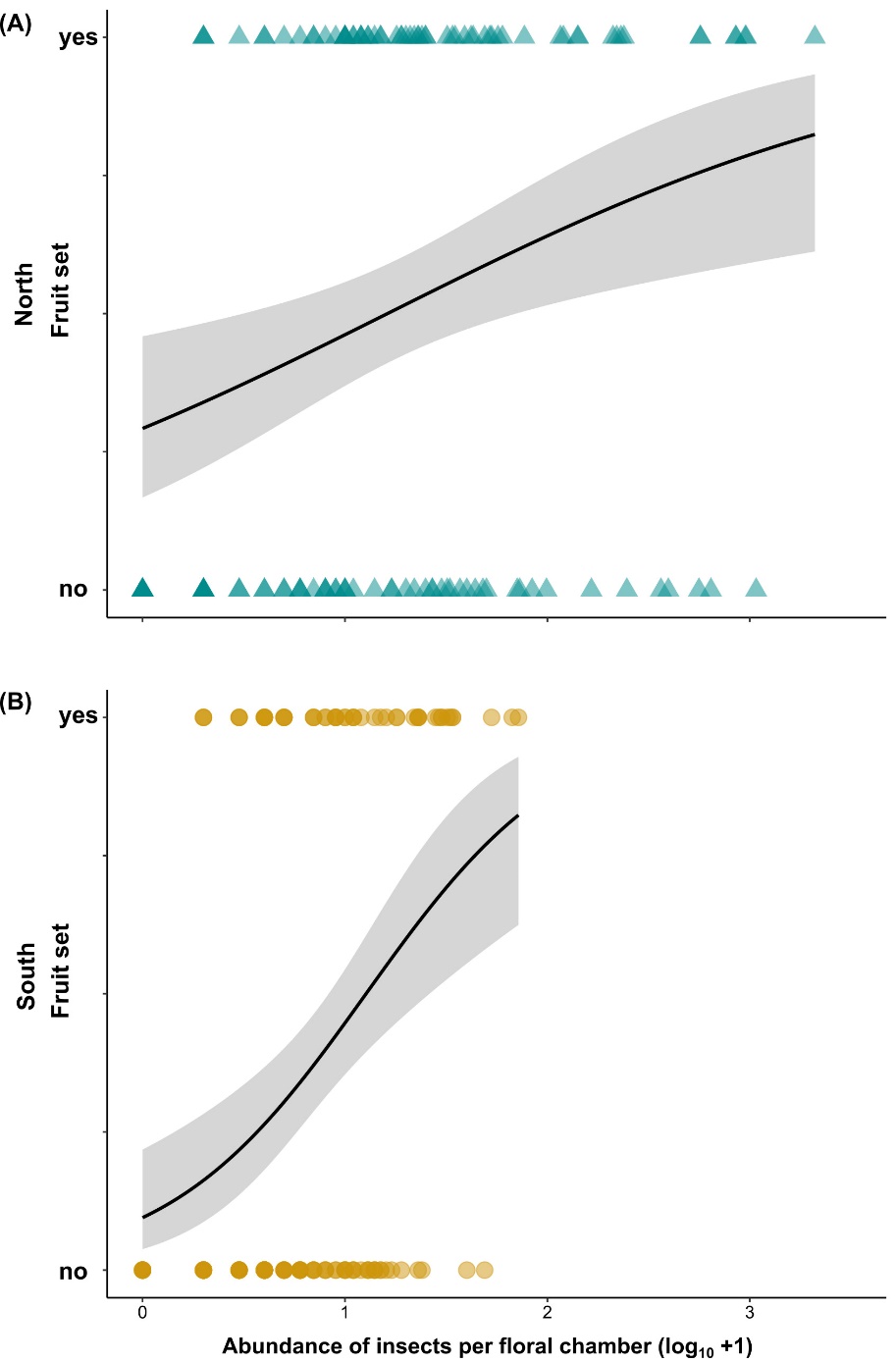


**Supplementary Figure 2.** Abundance of insects per floral chamber (log_10_ +1) of *Arum maculatum* individuals with and without an infructescence, (A) north and (B) south of the Alps. Logistic regressions indicate that the abundance of floral visitors is a significant predictor of the presence of an infructescence (black solid curves). Shaded areas depict the 95% confidence intervals around regression curves.

**
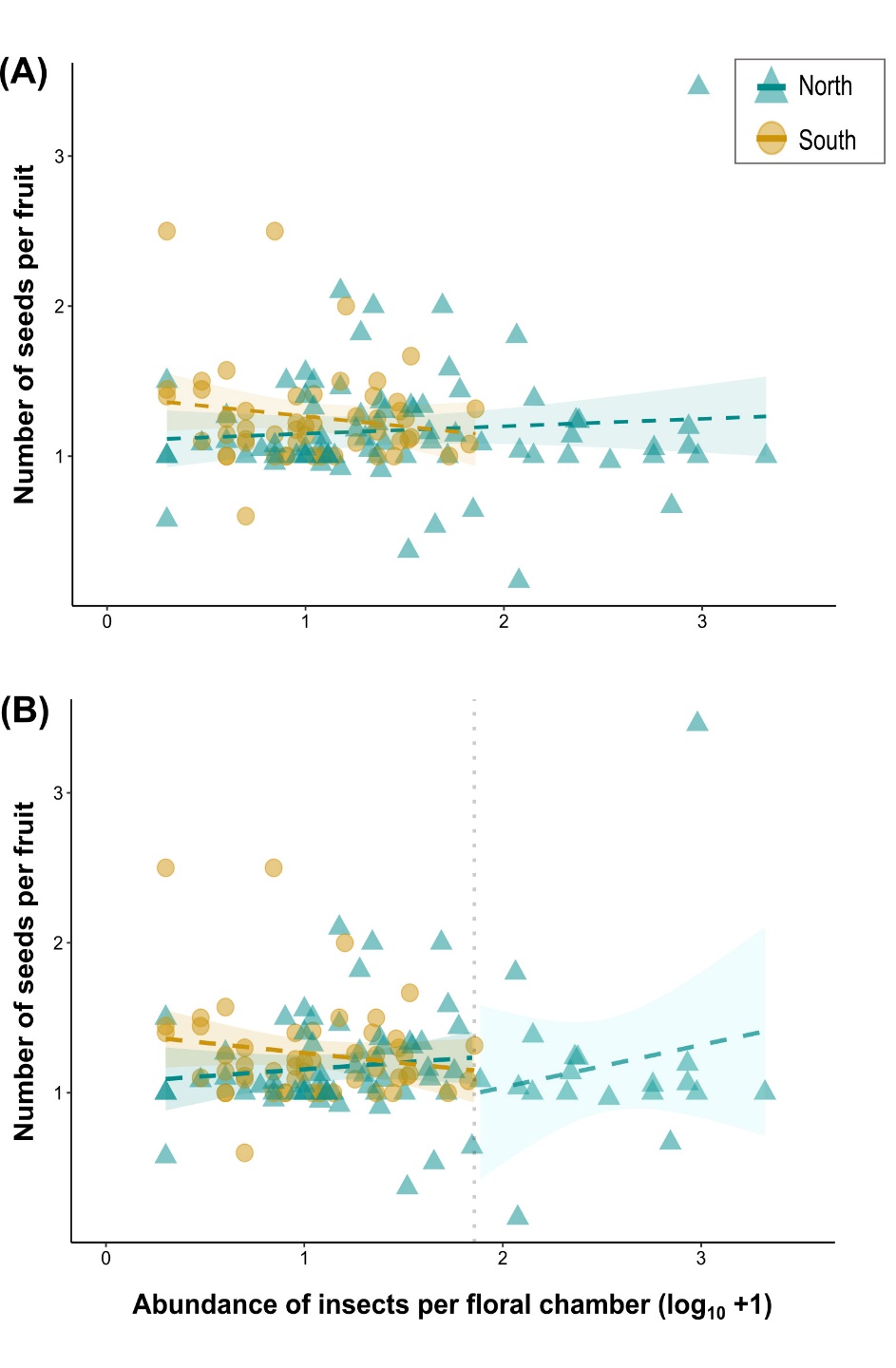
**

**Supplementary Figure 3.** (A) Linear regressions between the number of seeds per fruit of *Arum maculatum* individuals and absolute abundance (*log*_10_ + 1) of visitors per floral chamber for populations from north (blue lines/triangles) *vs*. south (dark yellow lines/circles) of the Alps. In (B) two linear regressions were calculated for the north; one based on floral chambers with a maximum of 71 visitors, i.e. the highest number observed in the south (vertical dotted line; see text for details), and the other based on floral chambers with more than 71 insects. Dashed lines indicate non-significant model fitting. Shaded areas indicate 95% confidence intervals around regression lines.


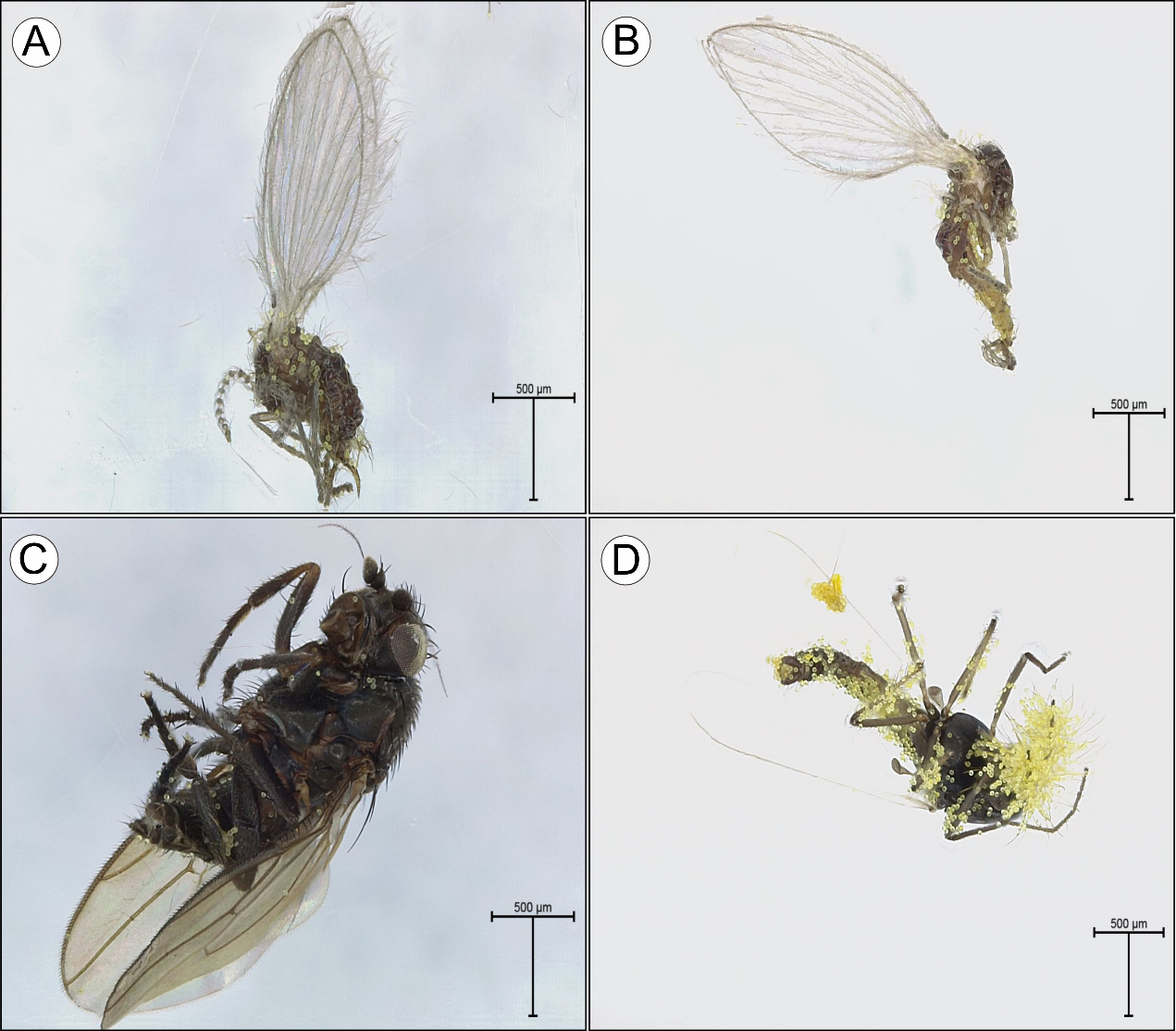


**Supplementary Figure 4.** Pollen grains of *Arum maculatum* observed on (A) female *Psychoda* sp*.,* (B) male *Psychoda* sp*.,* (C) Sphaeroceridae, and (D) Chironomidae.

## Supplementary Tables

**Supplementary Table 1.** Locations and year of sampling of populations of *Arum maculatum* from north vs. south of the Alps. N_FC_ and N_LT_ refer to the number of floral chambers sampled and light trap catches performed in each location, respectively.

| Region/population code | Locations | Country | Year of sampling | | | Latitude (N) | Longitude (E) |
| --- | --- | --- | --- | --- | --- | --- | --- |
|  |  |  | 2017 | 2018 | 2019 |  |  |
| North of the Alps |  |  |  |  |  |  |  |
| BUR | Burg Hohenstein, Hessen | D | - | *N*_FC_ = 16, *N*_LT_ = 1 | - | 50° 11.64’ | 8° 03.42’ |
| HOH | Hohendilching in Valley, Bavaria | D | *N*_FC_ = 8, *N*_LT_ = 1 | - | - | 47° 54.50’ | 11° 46.87’ |
| JOS | Josefiau, Salzburg | AT | *N*_FC_ = 53, N_LT_ = 2 | *N*_FC_ = 15 | *N*_FC_ = 3, *N*_LT_ = 1 | 47° 46.98’ | 13° 04.50’ |
| MUR | Murnau am Staffelsee, Bavaria | D | - | *N*_FC_ = 15, *N*_LT_ = 1 | - | 47° 40.20’ | 11° 10.80’ |
| NEC | Horb am Neckar, Baden-Württemberg | D | - | *N*_FC_ = 14, *N*_LT_ = 1 | - | 48° 25.20’ | 8° 39.00’ |
| RUM | Rümikon, Canton Aargau | CH | - | *N*_FC_ = 15, *N*_LT_ = 1 | - | 47° 31.00’ | 8° 21.00’ |
| South of the Alps |  |  |  |  |  |  |  |
| BER | Santa Maria Hoè, Lecco | IT | - | - | *N*_FC_ = 16, *N*_LT_ = 3 | 45° 44.90’ | 9° 22.00’ |
| DAO | Daone, Trentino | IT | *N*_FC_ = 62, *N*_LT_ = 2 | *N*_FC_ = 11, *N*_LT_ = 1 | - | 45° 57.60’ | 10° 34.80’ |
| LIM | Limone, Piemonte | IT | *N*_FC_ = 15, *N*_LT_ = 2 | - | - | 44° 12.60’ | 7° 34.20’ |
| MON | Montese, Modena | IT | *N*_FC_ = 15, *N*_LT_ = 1 | - | - | 44° 16.29’ | 10° 56.42’ |
| UDI | Casacco, Udine | IT |  | *N*_FC_ = 15, *N*_LT_ = 1 |  | 46° 10.59’ | 13° 15.55’ |

**Supplementary Table 2.** Mean fruit set (%) and absolute abundances of the collected insects per *Arum maculatum* population and region (north and south of the Alps) in floral chambers (FC) and light traps (LT; per hour of trapping). The median for each insect group and population across replicate floral chambers and light traps (if available) is shown in parentheses. ”*n*” refers to the number of floral chambers (FC) sampled in each population or to the number of light trap catches (LT) performed at each site. ”*N*” refers to the number of

infructescences found in each population. See **Supplementary Table** **S1** for identification of population codes.

|  | Northern populations | | | | | | | | | | | |
| --- | --- | --- | --- | --- | --- | --- | --- | --- | --- | --- | --- | --- |
|  | BUR | | HOH | | JOS | | MUR | | NEC | | RUM | |
|  | FC | LT | FC | LT | FC | LT | FC | LT | FC | LT | FC | LT |
|  | *n* = 16 | *n* = 1 | *n* = 8 | *n* = 1 | *n* = 71 | *n* = 3 | *n* = 15 | *n* = 1 | *n* = 14 | *n* = 1 | *n* = 15 | *n* = 1 |
| Fruit set (*N*)^1^ | 36.0 (8) | - | 86.1 (8) | - | 29.2 (26) | - | 34.8 (8) | - | 30.2 (5) | - | 50.9 (10) | - |
| Insect groups |  |  |  |  |  |  |  |  |  |  |  |  |
| Psychodidae |  |  |  |  |  |  |  |  |  |  |  |  |
| Males | 12 (0) | 4 | - | 5 | 46 (0) | 27 (3) | 20 (1) | 8 | - | 124 | 2 (0) | 13 |
| Females | 2036 (42.5) | 139 | 487 (21) | 322 | 1012 (7) | 161 (32) | 9939 (556) | 576 | 65 (4) | 28 | 704 (30) | 71 |
| *Psychoda brevicornis* | - | - | - | - | 1 (0) | 2 (0) | - | 4 | - | - | - | 1 |
| *P. grisescens* | 87 (1.5) | 17 | 4 (0) | 60 | 54 (0) | 72 (14) | 35 (1) | 64 | 8 (0.5) | 4 | 16 (1) | 15 |
| *P. phalaenoides* | 1850 (36.5) | 104 | 460 (18.5) | 119 | 832 (5) | 28 (6) | 9276 (524) | 400 | 47 (3) | 2 | 657 (27) | 28 |
| *P.* sp. 1^2^ | - | - | - | 37 | - | - | - | - | - | - | - | - |
| *P. trinodulosa* | 2 (0) | 5 | - | 7 | 10 (0) | 4 (1) | 72 (2) | 64 | - | - | 10 (0) | 9 |
| *P. zetterstedti* | 2 (0) | - | 4 (0) | 75 | 5 (0) | 14 (1) | 96 (2) | - | 1 (0) | 2 | - | 5 |
| Unidentified females | 95 (2.5) | 13 | 19 (1.5) | 24 | 110 (0) | 41 (12) | 460 (15) | 44 | 9 (0.5) | 20 | 21 (1) | 13 |
| Unknown sex | 1 (0) | - | - | - | 20 (0) | - | 34 (1) | 4 | - | 24 | 7 (0) | - |
| Other Diptera |  |  |  |  |  |  |  |  |  |  |  |  |
| Cecidomyiidae | - | - | - | 27 | 5 (0) | 39 (4) | - | 8 | 2 (0) | 28 | - | 13 |
| Ceratopogonidae | 245 (0.5) | 553 | 3 (0) | 13 | 54 (0) | 199 (19) | 45 (2) | 12 | 3 (0) | 42 | 4 (0) | 108 |
| Chironomidae | 20 (0.5) | 3 | - | 67 | 34 (0) | 83 (14) | 20 (0) | 24 | 11 (0) | 210 | 15 (0) | 313 |
| Sciaridae | 2 (0) | 1 | 3 (0) | 7 | 4 (0) | 16 (5) | 4 (0) | - | 5 (0) | 92 | 1 (0) | 8 |
| Sphaeroceridae | 3 (0) | - | - | 3 | 24 (0) | - | 42 (2) | - | 3 (0) | - | 5 (0) | - |
| Unidentified Diptera | - | - | - | 1 | 3 (0) | 12 (4) | - | - | 1 (0) | 6 | 1 (0) | - |
| Other Insecta |  |  |  |  |  |  |  |  |  |  |  |  |
| Coleoptera | 3 (0) | - | 1 (0) | - | 5 (0) | 1 (0) | 11 (0) | - | 1 (0) | 8 | 1 (0) | - |
| Hemiptera | - | - | 1 (0) | - | - | 3 (0) | - | - | - | - | - | - |
| Unidentified Insecta | - | - | - | 7 | 2 (0) | 2 (0) | - | - | - | 4 | - | 1 |
| **Total number of insects** | 2322 | 701 | 495 | 451 | 1209 | 543 | 10115 | 632 | 91 | 566 | 740 | 529 |

|  | Southern populations | | | | | | | | | | |
| --- | --- | --- | --- | --- | --- | --- | --- | --- | --- | --- | --- |
|  | BER | | DAO | | LIM | | MON | | UDI | | |
|  | FC | LT | FC | LT | FC | LT | FC | LT | FC | LT |  |
|  | *n* = 16 | *n* = 3 | *n* = 73 | *n* = 3 | *n* = 15 | *n* = 2 | *n* = 15 | *n* = 1 | *n* = 15 | *n* = 1 |  |
| Fruit set (*N*)^1^ | 7.1 (2) | - | 25.9 (32) | - | 41.6 (10) | - | 1.0 (1) | - | 21.6 (6) | - |  |
| Insect groups |  |  |  |  |  |  |  |  |  |  |  |
| Psychodidae |  |  |  |  |  |  |  |  |  |  |  |
| Males | 10 (0) | 1 (0) | 89 (0) | 2 (0) | 11 (0) | 3 (1.3) | 9 (0) | - | - | - |  |
| Females | 33 (1) | 2 (0) | 182 (1) | 17 (8) | 55 (3) | 7 (3.7) | 10 (0) | 6 | 27 (1) | - |  |
| *Psychoda brevicornis* | - | - | 4 (0) | - | 3 (0) | - | - | - | - | - |  |
| *P. grisescens* | 9 (0) | 1 (0) | 31 (0) | 8 (2) | 33 (2) | 1 (0.6) | 7 (0) | 5 | 21 (1) | - |  |
| *P. phalaenoides* | 20 (0) | - | 55 (0) | 6 (0.8) | 12 (0) | 3 (1.3) | - | - | - | - |  |
| *P.* sp. 1^2^ | - | - | 1 (0) | - | 4 (0) | - | - | - | - | - |  |
| *P. trinodulosa* | - | - | 6 (0) | 2 (0.8) | - | - | - | - | 1 (0) | - |  |
| *P. zetterstedti* | - | - | - | - | 1 (0) | 1 (0.6) | - | 1 | 1 (0) | - |  |
| Unidentified females | 4 (0) | 1 (0) | 85 (0) | 1 (0) | 2 (0) | 2 (1) | 3 (0) | - | 4 (0) | - |  |
| Unknown sex | 1 (0) | - | 78 (0) | - | 2 (0) | - | 3 (0) | - | - | - |  |
| Other Diptera |  |  |  |  |  |  |  |  |  |  |  |
| Cecidomyiidae | - | 33 (9.6) | - | 29 (7) | 1 (0) | 39 (19.5) | - | 19 | - | - |  |
| Ceratopogonidae | 5 (0) | 10 (4) | 8 (0) | 128 (4.8) | 6 (0) | 8 (4) | - | 2 | 3 (0) | - |  |
| Chironomidae | 3 (0) | 26 (9) | 105 (0) | 55 (11) | 29 (1) | 60 (29.8) | 6 (0) | 8 | 4 (0) | 38 |  |
| Sciaridae | 5 (0) | 13 (5) | 7 (0) | 71 (5) | 3 (0) | 10 (5) | - | 8 | 1 (0) | 10 |  |
| Sphaeroceridae | 10 (0) | 2 (1) | 494 (3) | 1 (0) | 68 (4) | 4 (1.9) | 3 (0) | 5 | 4 (0) | 2 |  |
| Unidentified Diptera | - | 5 (1) | 11 (0) | 4 (1) | 1 (0) | - | 2 (0) | 5 | 2 (0) | 8 |  |
| Other Insecta |  |  |  |  |  |  |  |  |  |  |  |
| Coleoptera | 1 (0) | 16 (0) | 3 (0) | - | 9 (0) | - | - | - | 2 (0) | - |  |
| Hemiptera | - | - | 1 (0) | 2 (0) | 1 (0) | - | 5 (0) | 1 | 2 (0) | - |  |
| Unidentified Insecta | 8 (0) | 6 (1) | 1 (0) | 3 (1) | - | 9 (4.6) | - | 1 | - | 2 |  |
| **Total number of insects** | 76 | 114 | 979 | 312 | 186 | 140 | 38 | 55 | 45 | 60 |  |

^1^ Data on the fruit set of some individuals are also presented in Gfrerer *et al.* (2021). Calculations are based on *N* = 260 plant individuals (see text for details).

^2^ *P.* sp. 1 refers to a specific morphotype of an unknown psychodid species.

**Supplementary Table 3.** Linear model testing for relationships between fruit set (%) of *Arum maculatum* individuals (response variable) and absolute abundances of different insect groups in logarithmic scale (explanatory variable) in the overlapping range of visitor abundances in northern (N) and southern (S) areas (1–71 flies per chamber; see also Figure 5). The adjusted *R^2^* and *p*-values refer to the performance and significance of the models, respectively. Significant values of the slopes (CI: confidence interval) are printed in bold. Asterisks (*) indicate significant differences (*p* < .05) in the intercept between the regions; fem.: female

| Insect groups | Region | adj. *R*^2^ (*p* value) | d.f. | Slope (CI) |
| --- | --- | --- | --- | --- |
| All insects (sum)* | N | 0.12 (< 0.001) | 99 | **28.93 (± 14.64)** |
|  | S | 0.18 (< 0.001) | 131 | **32.25 (± 11.68)** |
| Psychodidae | N | 0.14 (< 0.001) | 99 | **29.26 (± 14.04)** |
|  | S | 0.16 (< 0.001) | 131 | **36.30 (± 13.98)** |
| Non-Psychodidae* | N | 0.01 (0.141) | 99 | 18.97 (± 25.09) |
|  | S | 0.13 (< 0.001) | 131 | **25.46 (± 11.19)** |
| Males (Psychodidae)* | N | < 0.01 (0.445) | 99 | 19.43 (± 49.62) |
|  | S | 0.07 (0.001) | 131 | **33.81 (± 19.85)** |
| *P. phalaenoides* (fem.) | N | 0.13 (< 0.001) | 99 | **28.06 (± 13.92)** |
|  | S | 0.08 (< 0.001) | 131 | **40.93 (± 22.20)** |
| *P. grisescens* (fem.)* | N | 0.02 (0.087) | 99 | 30.06 (± 34.13) |
|  | S | 0.03 (0.018) | 131 | **28.68 (± 23.37)** |
| Ceratopogonidae* | N | < 0.01 (0.241) | 99 | 22.27 (± 36.98) |
|  | S | < 0.01 (0.145) | 131 | 33.55 (± 44.87) |
| Chironomidae* | N | < 0.01 (0.711) | 99 | 7.56 (± 39.89) |
|  | S | 0.08 (< 0.001) | 131 | **33.31 (± 18.52)** |
| Sphaeroceridae* | N | < 0.01 (0.989) | 99 | 0.31 (± 45.04) |
|  | S | 0.12 (< 0.001) | 131 | **24.71 (± 10.92)** |

# Supplementary Table 4. Results of restricted likelihood ratio (RLR) tests testing whether the variance for the term population as a random effect is zero (null hypothesis) for North [all individuals; considering only the ones with a maximum of 71 visitors (breakpoint)] and South of the Alps. *p* values were calculated based on 10,000 simulated values; significant values are marked in bold.

|  | RLR | *p* value |
| --- | --- | --- |
| North (all individuals) | 5.30 | **0.006** |
| North (before breakpoint) | 1.87 | 0.054 |
| South | 1.10 | 0.100 |

# 
